# Genome-Wide Screening for Enteric Colonization Factors in Carbapenem-Resistant ST258 Klebsiella pneumoniae

**DOI:** 10.1128/mBio.02663-18

**Published:** 2019-03-12

**Authors:** Hea-Jin Jung, Eric R. Littmann, Ruth Seok, Ingrid M. Leiner, Ying Taur, Jonathan Peled, Marcel van den Brink, Lilan Ling, Liang Chen, Barry N. Kreiswirth, Andrew L. Goodman, Eric G. Pamer

**Affiliations:** a Immunology Program, Sloan Kettering Institute, Memorial Sloan Kettering Cancer Center, New York, New York, USA; b Center for Microbes, Inflammation, and Cancer, Molecular Microbiology Core Facility, Memorial Sloan Kettering Cancer Center, New York, New York, USA; c Infectious Diseases Service, Department of Medicine, Memorial Sloan Kettering Cancer Center, New York, New York, USA; d Adult Bone Marrow Transplant Service, Department of Medicine, Memorial Sloan Kettering Cancer Center, New York, New York, USA; e Public Health Research Institute, New Jersey Medical School, Rutgers, the State University of New Jersey, Newark, New Jersey, USA; f Department of Microbial Pathogenesis and Microbial Sciences Institute, Yale University School of Medicine, New Haven, Connecticut, USA; Louis Stokes Veterans Affairs Medical Center

**Keywords:** *Klebsiella pneumoniae*, genome-wide screening, intestinal colonization, multidrug resistance, opportunistic infections

## Abstract

A diverse, antibiotic-naive microbiota prevents highly antibiotic-resistant microbes, including carbapenem-resistant Klebsiella pneumoniae (CR*-Kp*), from achieving dense colonization of the intestinal lumen. Antibiotic-mediated destruction of the microbiota leads to expansion of CR*-Kp* in the gut, markedly increasing the risk of bacteremia in vulnerable patients. While preventing dense colonization represents a rational approach to reduce intra- and interpatient dissemination of CR*-Kp*, little is known about pathogen-associated factors that enable dense growth and persistence in the intestinal lumen. To identify genetic factors essential for dense colonization of the gut by CR*-Kp*, we constructed a highly saturated transposon mutant library with >150,000 unique mutations in an ST258 strain of CR*-Kp* and screened for *in vitro* growth and *in vivo* intestinal colonization in antibiotic-treated mice. Stochastic and partially reversible fluctuations in the representation of different mutations during dense colonization revealed the dynamic nature of intestinal microbial populations. We identified genes that are crucial for early and late stages of dense gut colonization and confirmed their role by testing isogenic mutants in *in vivo* competition assays with wild-type CR-*Kp*. Screening of the transposon library also identified mutations that enhanced *in vivo* CR*-Kp* growth. These newly identified colonization factors may provide novel therapeutic opportunities to reduce intestinal colonization by CR-*Kp*.

## Abstract (precis)

Klebsiella pneumoniae is a common cause of bloodstream infections in immunocompromised and hospitalized patients, and over the last 2 decades, some strains have acquired resistance to nearly all available antibiotics, including broad-spectrum carbapenems. The U.S. Centers for Disease Control and Prevention has listed carbapenem-resistant K. pneumoniae (CR*-Kp*) as an urgent public health threat. Dense colonization of the intestine by CR*-Kp* and other antibiotic-resistant bacteria is associated with an increased risk of bacteremia. Reducing the density of gut colonization by CR*-Kp* is likely to reduce their transmission from patient to patient in health care facilities as well as systemic infections. How CR*-Kp* expands and persists in the gut lumen, however, is poorly understood. Herein, we generated a highly saturated mutant library in a multidrug-resistant K. pneumoniae strain and identified genetic factors that are associated with dense gut colonization by K. pneumoniae. This study sheds light on host colonization by K. pneumoniae and identifies potential colonization factors that contribute to high-density persistence of K. pneumoniae in the intestine.

## INTRODUCTION

Klebsiella pneumoniae is a leading cause of infections, including pneumonia, bacteremia, urinary tract infection, and liver abscess (1). It is also one of the most commonly isolated Enterobacteriaceae species causing infections in cancer patients and has been associated with high mortality (2). Treatment of K. pneumoniae infection can be challenging due to its broad antibiotic resistance. Since the initial isolation in 1996 of a K. pneumoniae strain resistant to carbapenems—a class of broad-spectrum antibiotics that is generally reserved for treatment of highly antibiotic-resistant bacterial infections (3)—the frequency of carbapenem resistance among clinical *Klebsiella* isolates increased to 1.6% in 2001 and 10.4% in 2011 (4). Given the limited repertoire of antibiotics that can be used to treat carbapenem-resistant K. pneumoniae (CR*-Kp*), efforts to limit infections have focused on reducing transmission within health care settings. A recent study by Snitkin et al. (5) demonstrated that CR*-Kp* is readily transmitted from patient to patient and that resistant strains can cause regional outbreaks when patients are transferred from one institution to another. Although patient isolation, gloving, gowning, and vigorous handwashing reduce transfer of highly antibiotic-resistant pathogens between patients, high-density shedding of these pathogens in patient feces renders standard infection control strategies less than fully effective (4, 6).

Treatment of cancer with cytotoxic chemotherapy and/or hematopoietic stem cell transplantation often requires treatment with antibiotics, which can alter the intestinal microbiota and disrupt “colonization resistance” (7, 8). This circumstance enables opportunistic pathogens like K. pneumoniae to expand and densely colonize the gut (9). Compromised immune defenses in patients undergoing cancer treatment also contribute to dense colonization of the gut by antibiotic-resistant bacteria (10). Disruption of the mucosal surface by neoplasms or development of mucosal inflammation secondary to cancer treatments predisposes patients to systemic infections, including bacteremia, with antibiotic-resistant pathogens that have colonized the intestine at a high density (9, 11, 12). Dense colonization of the gut by pathogens also contributes to patient-to-patient transmission in health care settings (13). Although colonization of the intestine by antibiotic-resistant bacteria, including CR*-Kp*, has been demonstrated in mouse models to be dependent on antibiotic-mediated abrogation of colonization resistance, little is known about bacterial factors that enable rapid growth and high-density persistence in the gut lumen.

Transposon mutant libraries have been used to identify genes that are involved in host colonization and pathogenesis of infection and genome-wide transposon insertion sequencing has facilitated the identification of new, unsuspected mechanisms of pathogenesis (14, 15). In this study, we use insertion sequencing (INSeq) (16) to identify genetic factors that contribute to dense colonization of the gut by the carbapenem-resistant ST258 strain of K. pneumoniae. Recent studies using mouse models demonstrated that host defenses against clinical isolates of K. pneumoniae differ from those previously documented for the most frequently studied, rodent-adapted K. pneumoniae strain ATCC 43816 (17, 18). Given the clinical challenges posed by CR-*Kp* infections, we opted to use an ST258 strain isolated from a blood culture of a patient with K. pneumoniae bacteremia (18) to construct a transposon mutant library. *In vivo* screening of the transposon library identified genes that facilitated short- and long-term, high-density colonization of the intestinal tract, and their role was confirmed by the generation and complementation of isogenic mutants.

## RESULTS

### Dense colonization of the gut by K. pneumoniae and development of K. pneumoniae bacteremia.

**FIG 1:**
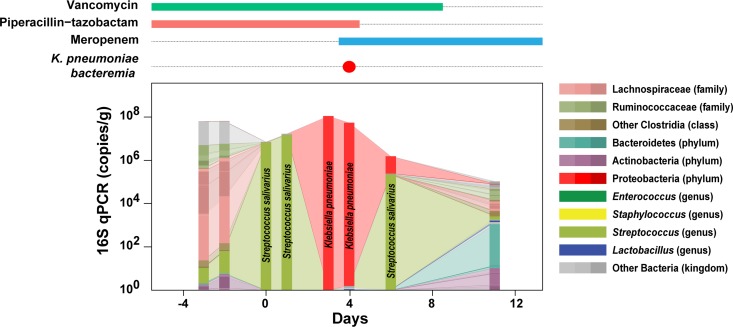
Dense colonization of the intestine by K. pneumoniae preceded K. pneumoniae bacteremia. Changes in the gut microbiota of an allo-HCT patient who developed K. pneumoniae bacteremia were characterized by 16S rRNA sequencing and qPCR of stool samples. Sample collection days are indicated relative to the day of transplant (day 0). Concurrent intravenous antibiotic treatment and detection of bacteremia are shown at the top.

Previous studies demonstrated that dense colonization of the intestine by vancomycin-resistant *Enterococcus* (VRE) is associated with VRE bacteremia and dense colonization by proteobacteria is associated with Gram-negative rod bacteremia (11, 12). We performed a longitudinal 16S rRNA sequence analysis on fecal samples obtained from a patient undergoing allogeneic hematopoietic cell transplantation (allo-HCT) who developed bacteremia with a carbapenem-sensitive K. pneumoniae strain (Fig. 1). Prior to stem cell infusion (day 0), the patient’s fecal microbiota was diverse. However, as the treatment proceeded, many bacterial taxa were lost, and after transient domination by Streptococcus salivarius, the patient was densely colonized by K. pneumoniae on the 3rd and 4th days following stem cell infusion. The marked expansion of fecal K. pneumoniae was followed by bacteremia; treatment with meropenem led to rapid clearance of K. pneumoniae from blood and feces. This longitudinal analysis of microbiota changes in an allo-HCT patient demonstrates the remarkable ability of K. pneumoniae to rapidly expand and achieve bacterial densities, as a major bacterial inhabitant, of 10^8^ 16S rRNA gene copies/gram of feces.

### A highly saturated transposon mutant library was generated in multidrug-resistant ST258 K. pneumoniae.

To identify genes involved in dense colonization of the intestine, we pursued a genome-wide high-throughput screening approach using a transposon mutant library in a multidrug-resistant ST258 K. pneumoniae isolate, MH258, which came from another patient (18). Given the clinical challenges posed by CR-*Kp* infections and the possibility that there may be differences between strains in terms of gut colonization in comparison with previous studies, we opted to use a carbapenem-resistant ST258 strain rather than the carbapenem-sensitive K. pneumoniae strain shown in Fig. 1. Generation of this library in a highly antibiotic-resistant bacterial strain presented technical challenges (see Fig. S1 in the supplemental material; see also Text S1 posted at doi.org/10.6084/m9.figshare.7063823 and Materials and Methods), but we obtained more than 300,000 distinct colonies to generate the mutant library. A reference genome for sequence alignment was prepared by fully sequencing MH258 on PacBio and Illumina platforms. The MH258 genome is composed of a chromosome of ∼5.55 Mb and three plasmids with sizes of 208, 78, and 43 kb; based on the annotation by PATRIC (19), the chromosomal DNA encodes 5,473 coding sequences (CDSs) (see Table S1 posted at doi.org/10.6084/m9.figshare.7063823).

10.1128/mBio.02663-18.1 FIG S1 Antibiotic screening to identify potential selection markers for strain MH258. (A) Disk diffusion assay with E. coli DH5α pDB60, E. coli S17-1 λpir pSAM (16), MH258, and E. coli S17-1 λpir strains. The kinds and amounts of antibiotics loaded on each filter disk are noted. Strain MH258 appeared to be sensitive to streptomycin, rifampin, and gentamicin. It was also sensitive to tetracycline, but to a limited extent. Amp, ampicillin; Chlr, chloramphenicol; Gen, gentamicin; Kan, kanamycin; Spec, spectinomycin; Strep, streptomycin; Tet, tetracycline; Car, carbenicillin; Clin, clindamycin; Ery, erythromycin; Met, metronidazole; Rif, rifampin; Van, vancomycin. (B) Antibiotic screening with multiple K. pneumoniae strains. Cultures of multiple K. pneumoniae strains (18) and reference E. coli strains were dotted on LB plates containing gentamicin (10 μg/ml), streptomycin (50 μg/ml), or tetracycline (10 μg/ml) and incubated overnight at 37°C. For MH258, two distinct clones were tested. All the K. pneumoniae strains barely grew on plates with gentamicin or streptomycin; MH258 grew fairly well on a tetracycline plate. (C) Disk diffusion assay to confirm that the pDB60 plasmid does not confer resistance to amikacin, gentamicin, and tobramycin—all clinically relevant antibiotics to treat carbapenem-resistant K. pneumoniae. Download FIG S1, PDF file, 1.5 MB. Copyright © 2019 Jung et al. 2019 Jung et al. This content is distributed under the terms of the Creative Commons Attribution 4.0 International license.

**FIG 2:**
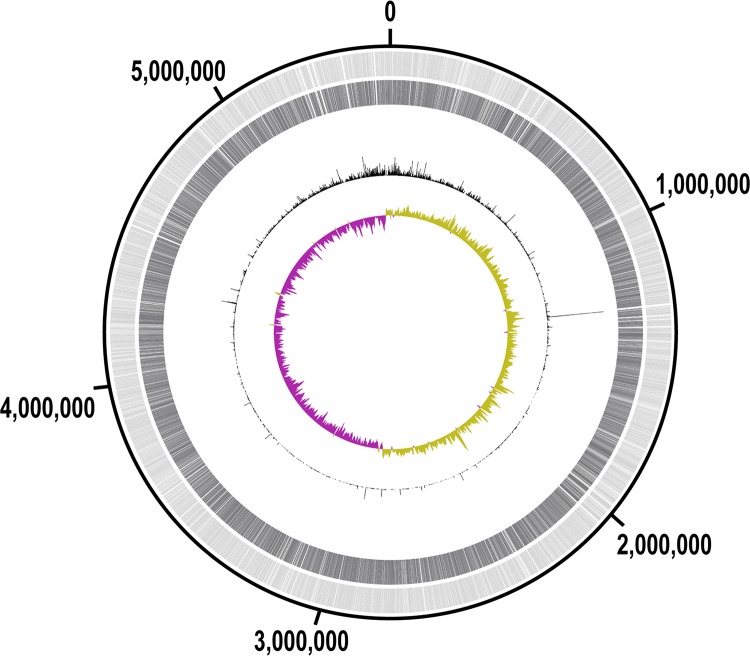
A transposon mutant library was constructed in K. pneumoniae MH258. Distribution of transposon mutants in the MH258 genome. Track 1 (light gray, outermost) shows all coding sequences (CDSs) in the chromosomal DNA. Track 2 (dark gray) shows the CDSs in which at least one mutant was found in the library. Track 3 (black peaks, middle) shows read counts for each insertion site. Track 4 (yellow-green/purple, innermost) shows a GC skew plot using a sliding window size of 10 kb (yellow-green, above average; purple, below average). The plot was generated using DNAPlotter (49).

About 6 million reads from the mutant library were aligned to the K. pneumoniae MH258 genome and identified ∼150,000 unique transposon mutants (Fig. 2). This covers 76% of all the potential TA insertion sites (151,656/198,684 TA sites) and 99.9% of all the CDSs in the chromosomal DNA (5,469/5,473 CDSs). The mutations were distributed across the entire chromosome (Fig. 2)—which, together with the high coverage rate, is well suited for genome-wide screening. A slight bias toward the origin of replication was observed (Fig. 2), as expected for a rapidly growing bacterial strain.

While most of the CDSs were associated with at least one mutant in the library, the read counts for some genes were significantly lower than others (Fig. S2), suggesting that those genes may be essential. Detection of mutations in essential genes likely resulted from pooling of medium-flooded plates (see Materials and Methods), which incorporates near-static and slowly growing cells resulting from mutations in essential genes. Reduction of incubation times for transposant selection to prevent overrepresentation of mutants with growth advantages also resulted in higher representation of defective mutants. Detection of silent mutations (e.g., mutations in the 3′ ends of genes) likely also contributed to the high CDS coverage. To further explore this, we determined gene essentiality using the EL-ARTIST pipeline (20). After a hidden Markov model (HMM) refinement, ∼14% of the chromosomal CDSs (776/5,473) were identified as essential (see Table S1 posted at doi.org/10.6084/m9.figshare.7063823). As we speculated, the genes with minimal read counts were designated as essential; in contrast, nonessential genes had many transposon insertions (Fig. S2).

10.1128/mBio.02663-18.2 FIG S2 Read counts for each insertion site in the genomic regions with essential and nonessential genes. Multiple mutants were detected in the nonessential genes (sky blue), but the essential genes (purple) were virtually devoid of mutants. The *y*-axis scales are 0 to 500, cutting off read counts over 500. The base positions are marked at the top. Gene names were assigned by BlastKOALA (51), and the plots were generated using Artemis (52). Download FIG S2, PDF file, 0.4 MB. Copyright © 2019 Jung et al. 2019 Jung et al. This content is distributed under the terms of the Creative Commons Attribution 4.0 International license.

### The mutant library was screened for *in vitro* growth and dense intestinal colonization in mice.

**FIG 3:**
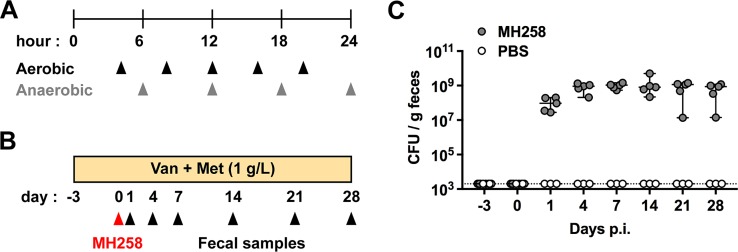
The mutant library was screened *in vitro* and *in vivo* to determine genetic factors that are specifically associated with gut colonization. (A) Experimental design of *in vitro* screening. To screen for defects in aerobic or anaerobic growth, the mutant libraries were sampled over 24 h as cultures reached early stationary phase and were passaged (arrowheads). (B) Experimental design of *in vivo* screening. Mice were treated with vancomycin (Van) and metronidazole (Met) for 3 days and inoculated with the mutant library (∼10^8^ CFU in 200 μl PBS) by oral gavage. Fecal samples were collected thereafter for 4 weeks. (C) Colonization levels of the mutant library in the inoculated mice (*n *=* *5). Fecal samples were suspended in PBS, and serial dilutions were plated on mutant-selective plates to enumerate CFU. PBS-treated control mice (*n *=* *3) were also evaluated to confirm the absence of cross-contamination as well as efficacy of the selective plates. Values are medians ± 95% confidence intervals. The dotted line marks the limit of detection.

We screened the mutant library *in vitro* to identify mutants with general growth defects. During gut colonization, bacteria encounter aerobic and anaerobic conditions—aerobic until they reach to the lower gastrointestinal tract and anaerobic in the cecum and colon. Therefore, we tested the effects of mutations on growth kinetics *in vitro* under aerobic and anaerobic conditions (Fig. 3A). To promote continuous growth, we inoculated ∼10^8^ CFU of the mutant library and passaged cultures into fresh media as they reached early stationary phase. Over 24 h, aerobic cultures were passaged 5 times, while anaerobic cultures were passaged 4 times—corresponding to more than 50 generations.

**FIG 4:**
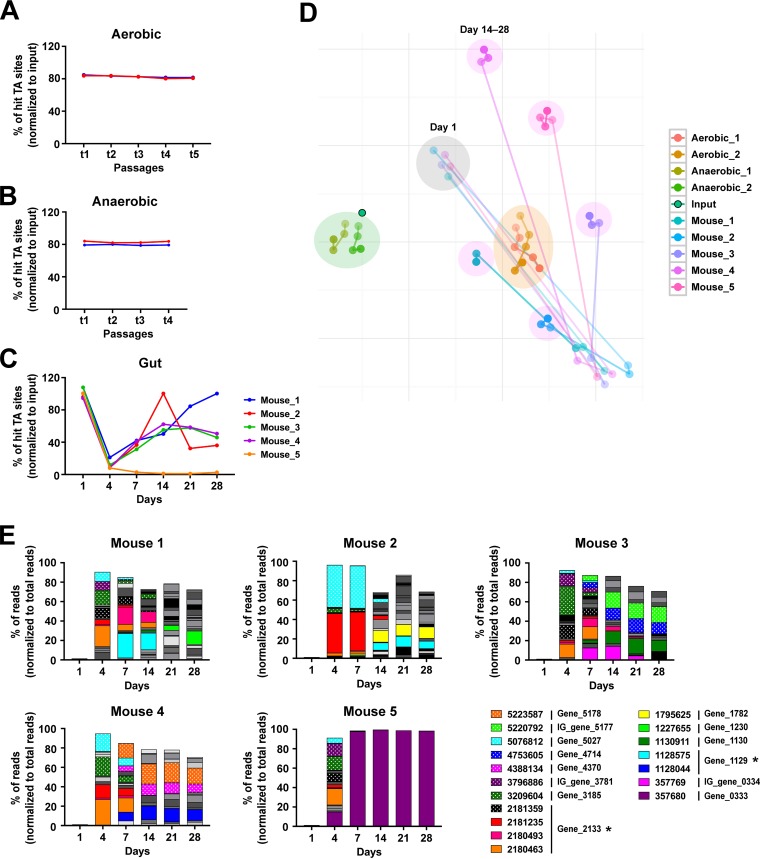
The mutant population greatly fluctuated in the gut with temporal expansion of a subset of mutants with enhanced fitness, but not *in vitro*. (A to C) Graphs showing changes in the diversity of the mutant populations during aerobic growth (A), anaerobic growth (B), and gut colonization (C). Sequencing depths were normalized to the lowest value, and the percentage of total targeted TA sites that were detected in each sample was plotted. (D) t-Stochastic neighbor embedding (t-SNE) was used to reduce the dimensionality of the data and visualize the clustering of the total mutant composition during gut colonization and *in vitro* growth. Samples collected longitudinally from each *in vitro* culture or mouse are presented in color—the darker color denotes the later time point. The clusters of *in vitro* aerobic and anaerobic samples are marked with orange and green background circles, respectively. The cluster of day 1 *in vivo* samples is highlighted with a gray circle, and day 14 to 28 sample clusters for each mouse are indicated in magenta. (E) Stacked graphs of percentages of reads from the mutants that showed enhanced fitness in the gut. All the mutants representing >2% of the total reads in any samples were graphed; mutants with >10% of the total reads were colored. Insertion sites of the mutants are noted in the legend. The genes for which isogenic mutants were generated and tested for a competitive colonization are marked with asterisks.

To screen the mutant library for genes that are required for dense intestinal colonization *in vivo*, we first administered vancomycin and metronidazole to mice for 3 days—thereby disrupting the normal microbiota and facilitating dense colonization of the gut lumen by K. pneumoniae, similar to the scenario found in hospitalized patients (Fig. 1 and 3B). After the mice were treated with antibiotics, they were inoculated with ∼10^8^ CFU of the mutant library by oral gavage. This dose contained approximately 10^2^ to 10^3^ CFU of each transposon mutant (10^8^ CFU/1.5 × 10^5^ mutants in the library), and as shown below (Fig. 4C), it allowed delivery of the entire library to the intestine of the recipient mouse through potential bottlenecks en route to the colon (21). We postulated that distinct sets of genes contribute to the initial colonization and persistence. To test this idea, we longitudinally collected fecal samples from colonized mice for 4 weeks (Fig. 3B). The density of gut colonization by MH258 mutants was maximal by day 4 postinoculation and remained high for the duration of the study (Fig. 3C).

### Mutant populations remained stable during *in vitro* culture but fluctuated during intestinal colonization, with temporal expansion of a subset of mutants with enhanced fitness.

To identify mutations that reduced or potentially enhanced fitness, we sequenced each sample, obtaining >2 million reads. Alignment of reads to the MH258 genome revealed that mutant populations were generally stable over the entire time of growth under *in vitro* aerobic and anaerobic culture conditions (Fig. 4A, B, and D). In contrast, during *in vivo* colonization, the diversity of the mutant populations was markedly reduced at day 4 and then gradually increased over the subsequent 10 days and stabilized (Fig. 4C and D). This finding supports our initial hypothesis that the mechanisms facilitating initial colonization with K. pneumoniae and subsequent persistence may be distinct. We also found that some mutants were highly enriched in fecal samples and that the population of enriched mutants fluctuated greatly on days 4 and 7 but then became more stable on day 14 (Fig. 4E and Fig. S3; see also Table S2 posted at doi.org/10.6084/m9.figshare.7063823). We did not detect enrichment of any mutants beyond 2% of the total reads either *in vivo* on day 1 or in the *in vitro* samples (see Table S2 posted at doi.org/10.6084/m9.figshare.7063823). For fecal samples obtained on days 4 and 7, as few as 1 to 13 mutants represented >75% of the total reads, each occupying at least 2% (see Table S2 posted at doi.org/10.6084/m9.figshare.7063823). For gene_0333 (*malT*), gene_1129 (encoding a formate hydrogenlyase transcriptional activator), gene_2133 (encoding a tail-specific protease precursor), and gene_5382 (*thuR*), multiple mutants with distinct insertions had enhanced fitness (Fig. S4A to D). Mutants in the genes that are involved in DNA mismatch repair (gene_0966, *mutH*; gene_1103, *mutS*) and maltose metabolism (gene_0333, *malT*; gene_4274, *malK*; gene_5177, *malE*; gene_5178, *malF*; gene_5179, *malG*) were also enriched, implying costs of those pathways in dense colonization of the intestine. We also saw enrichment of bacteria with a transposon insertion in gene_2182 (encoding a protein containing domains DUF403), gene_3185 (encoding a phage protein), or gene_5027 (*kefA*); however, with this group, enhanced fitness resulted from a single insertion site per gene (Fig. S4E to G). A *malT* mutant (gene_0333; with an insertion at 357680) completely dominated the gut of one mouse after day 7; however, this did not occur in the other four mice (Fig. 4E).

10.1128/mBio.02663-18.3 FIG S3 Some mutants were highly enriched in the mouse gut. Stacked graphs of percentages of reads for the genetic loci (genes and intergenic regions) whose mutants showed enhanced fitness. All the mutants that represented more than 2% of the total reads in any samples were accounted (Fig. 4E), and reads from the enriched mutants were grouped for each loci and graphed. Loci that represented >10% of the total reads were colored. Download FIG S3, PDF file, 0.6 MB. Copyright © 2019 Jung et al. 2019 Jung et al. This content is distributed under the terms of the Creative Commons Attribution 4.0 International license.

10.1128/mBio.02663-18.4 FIG S4 Percentages of reads for the mutants in gene_0333 (A), gene_1129 (B), gene_2133 (C), and gene_5382 (D), in which multiple mutants were enriched during gut colonization, and in gene_2182 (E), gene_3185 (F), and gene_5027 (G), where a single-insertion-site mutant was enriched. Read counts for each mutant were normalized to the total read counts and plotted as percentages. The *y*-axis scales are 0 to 0.1, cutting off the values over 0.1. Download FIG S4, PDF file, 0.7 MB. Copyright © 2019 Jung et al. 2019 Jung et al. This content is distributed under the terms of the Creative Commons Attribution 4.0 International license.

### Genetic factors specifically associated with dense intestinal colonization.

Expansion of a limited subset of mutants in fecal samples posed two major challenges for discovery of genetic regions that are essential for dense intestinal colonization. First, massive expansion of a subset of mutants can lead to apparent loss of low-abundance mutants that fall below the limit of detection, resulting in significant variation in library complexity between samples and incorrect assignment of essentiality. To correct for this, we adopted the ARTIST pipeline that simulates stochastic variability in output samples and generates normalized input data sets for Mann-Whitney U (MWU) analysis (20). Second, to minimize false-positive calls, positional bias of the mutant library should be normalized; for normalization, the local read counts are scaled to the total read counts using a sliding window (20). However, if there are insertion sites with very high read counts, normalization will render surrounding regions underrepresented. Meanwhile, silent mutations (e.g., mutations in the 3′ ends of genes) can neutralize the impact of other significant mutations in the same genes, as significance of each gene is determined by combining changes in all the disrupted sites within the given gene, thereby leading to the exclusion of true-positive calls. For these reasons, read filtering was essential; however, read filtering in data analysis of transposon insertion sequencing has been empirical and frequently varies from study to study. Therefore, we tested 18 read-filtering conditions to compare their impact on gene essentiality assignments (Fig. S5A; see also supplemental methods, Text S2, posted at doi.org/10.6084/m9.figshare.7063823 for details). In the case of the *in vitro* samples, the final outputs of MWU analysis (20) were similar except in the four conditions that filtered reads from mutants whose read counts from the left and right sides of insertion sites are more than 10-fold different (named “LRfold”) (Fig. S5B). However, when the mutant population was severely skewed, as observed in the fecal samples with highly enriched mutants, the different filtering conditions led to markedly different results. The numbers of significant genes varied from 43 in “Edit_Over_2” condition to 199 in “Over_10_Min_B” condition; some genes that were assigned as essential in other conditions (e.g., gene_0046) were not identified in the “Over_10_Min_B” condition with the highest number of significant genes (Fig. S5C).

10.1128/mBio.02663-18.5 FIG S5 Impacts of different read filterings on final MWU outputs. (A) Schematic of read filterings prior to the MWU analysis (20). Mutants with one of the following conditions were considered to be excluded: representing more than 10 (or 2) percentage of the total reads [> 10(2)% total reads]; with less than 2 read counts (< 2 reads); whose read counts from the left and right sides of insertion sites are more than 10-fold different (> 10-fold diff. in L/R); and located in the 3′ end (10%) of genes (10% 3′-end). For normalization of positional bias, two different window sizes (100,000-nt and 500,000-nt) were tested (Window size). The filtering criteria are noted at the top, and the chart is marked with O or X to indicate whether each criterion was applied or not applied for a given condition. For example, in “Over_10_Min” condition, reads from the mutants representing more than 10% of the total reads, reads with less than 2 read counts, or reads located in the 3′ end of genes were removed before normalization of positional bias using a window size of 100,000 nt. Reads from the mutants whose read counts from the left and right sides of insertion sites are more than 10-fold different were not removed, and after the positional bias normalization, no further filtering (< 2 reads; 10% 3′-end) was applied prior to the ARTIST MWU analysis. Please also see supplemental methods, Text S2, posted at doi.org/10.6084/m9.figshare.7063823 for further details. (B) Heat map of average log_2_ fold changes in the normalized read counts for final aerobic samples (aerobic_t5) with different read filterings. All the significant genes in any of the filtering conditions are listed on the *y* axis. Fold changes for the genes that were not significant in a given filtering condition are not shown; they were treated as unchanged (white). (C) Heat map of average log_2_ fold changes in the normalized read counts for day 28 fecal samples with different read filterings. Unlike aerobic_t5 samples shown in panel B, gene significances and fold changes were greatly affected by read filtering conditions. Download FIG S5, PDF file, 2.4 MB. Copyright © 2019 Jung et al. 2019 Jung et al. This content is distributed under the terms of the Creative Commons Attribution 4.0 International license.

**FIG 5:**
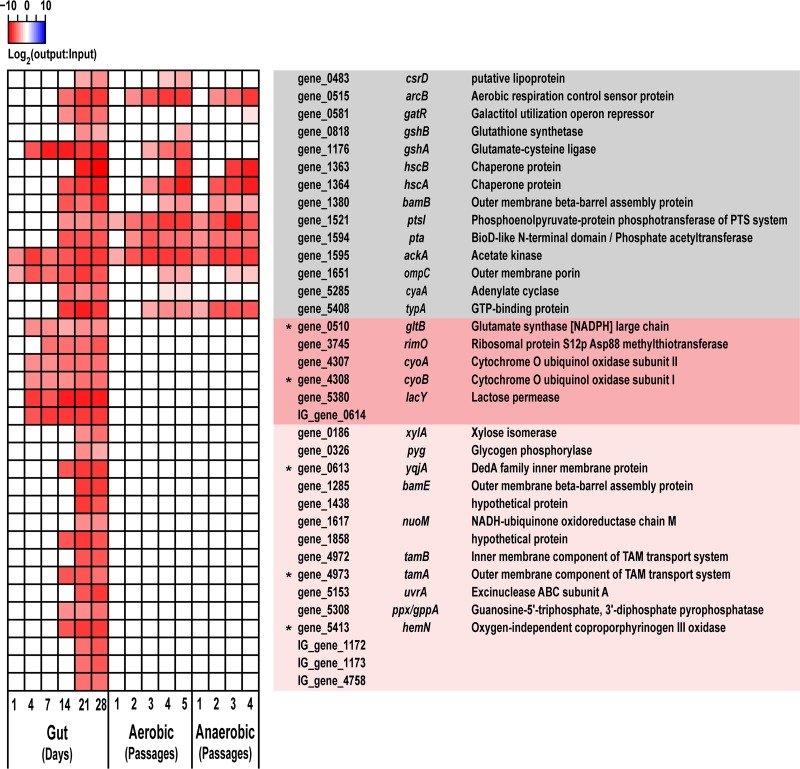
Genetic factors that are linked to dense intestinal colonization and *in vitro* growth were revealed. Each sample (from each animal at a given time point) was analyzed using the ARTIST pipeline, and the genetic loci whose mutants persistently had reduced fitness in all the examined animals are listed here. Average log_2_ fold changes in the normalized read counts for the top significant loci in intestinal colonization are shown as a heatmap on the left. The loci are color coded on the right depending on their *in vivo* and *in vitro* importance: gray, required both *in vivo* and *in vitro*; dark pink, required only *in vivo* from early colonization; light pink, required only *in vivo* at the later stages of colonization. The fold changes for the loci that were not significant in a given sample are not shown; they were treated as unchanged (white). The genes for which isogenic mutants were generated and tested for a competitive colonization are marked with asterisks.

To pinpoint the most relevant genes with higher confidence, we listed genes that were detected as significant under multiple filtering conditions in all the examined animals (see Table S3 posted at doi.org/10.6084/m9.figshare.7063823). We identified 35 loci (31 genes and 4 intergenic regions) whose mutants persistently had reduced fitness during dense colonization of the intestine (Fig. 5). Among these 35 loci, many were involved in energy metabolism and protein transport/folding and, in addition to being essential for *in vivo* persistence, were also required for *in vitro* growth. The other 21 loci (17 genes and 4 intergenic regions) were specific for gut colonization. Mutations in gene_0510 (*gltB*), gene_3745 (*rimO*), gene_4307 (*cyoA*), gene_4308 (*cyoB*), gene_5380 (*lacY*), and IG_gene_0614 (the intergenic region upstream of *yqjA*) resulted in reduced fitness as early as days 4 or 7, while other loci became essential around days 14 to 21. The full lists of genes associated with persistent changes *in vivo* or *in vitro* using a lower cutoff are provided in Fig. S6.

10.1128/mBio.02663-18.6 FIG S6 Genes associated with persistent changes *in vivo* or *in vitro* using a lower cutoff. Average log_2_ fold changes in the normalized read counts for the significant loci in intestinal colonization (A) and *in vitro* growth (B) are shown as heatmaps. All the loci that were detected as significant under at least 6 different filtering conditions (instead of 12 out of 18 conditions for Fig. 5) are listed. The loci are color coded on the right depending on their *in vivo* and *in vitro* importance. In panel A, the same color scheme as in Fig. 5 was applied. In panel B, the following colors were used: gray, required in all *in vivo* and *in vitro* conditions; green, required for both aerobic and anaerobic *in vitro* growth but not *in vivo*; yellow, required for aerobic, but not anaerobic, *in vitro* growth; magenta, required for anaerobic, but not aerobic, *in vitro* growth. Download FIG S6, PDF file, 1.9 MB. Copyright © 2019 Jung et al. 2019 Jung et al. This content is distributed under the terms of the Creative Commons Attribution 4.0 International license.

### Significance of the identified genes in gut colonization was confirmed using isogenic mutants.

**FIG 6:**
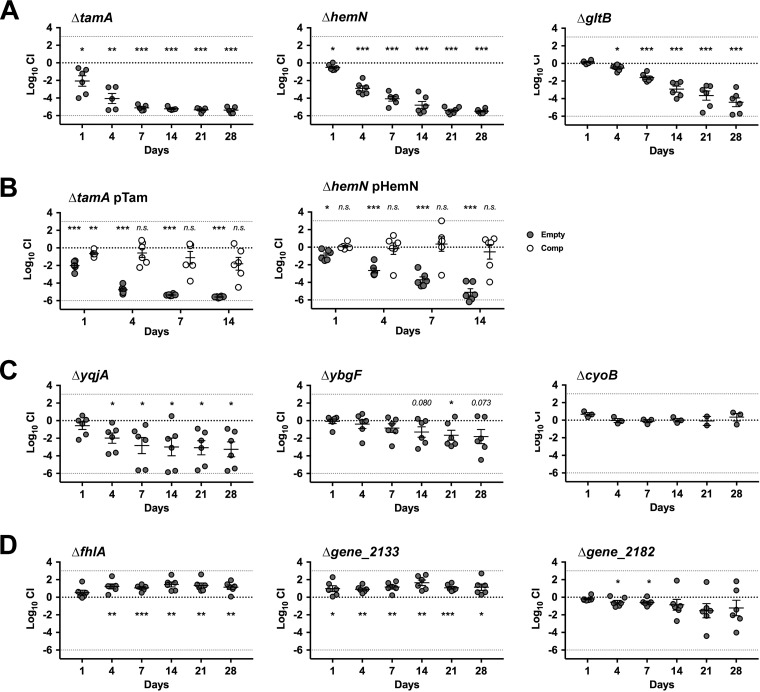
Competitive colonization study with isogenic mutants confirmed significance of the genes identified by INSeq in gut colonization. Nine isogenic mutants for the selected genes were generated by replacing the entire open reading frame (ORF) with a rifampin resistance cassette and tested for colonization of antibiotic-treated mice in competition with a wild-type strain. (A) Mutants with dramatic defects in gut colonization. (B) Complementary strains of Δ*tamA* and Δ*hemN* mutants were similarly tested with a wild-type strain harboring an empty pACYC177_aadA plasmid. Closed circles (Empty), Δ*tamA* pACYC177_aadA (or Δ*hemN pACYC177_aadA)* versus WT pACYC177_aadA; open circles (complementary [Comp]), Δ*tamA* pTam (or Δ*hemN pHemN)* versus WT pACYC177_aadA. (C) Mutants with minor or no defects in gut colonization. Δ*yqjA* and Δ*ybgF* mutants showed minor but significant defects, whereas the Δ*cyoB* mutant did not show fitness change. (D) Mutants with enhanced fitness. The Δ*flhA* and Δ*gene_2133* mutants showed enhanced fitness as predicted. The Δ*gene_2182* mutant showed a minor defect. Mean competitive index (CI) ± SEM for each mutant is shown on a log scale. Statistical significance by one-sample *t* test is shown by asterisks as follows: *, *P* < 0.05; **, *P* < 0.01; ***, *P* < 0.001.

To confirm essentiality of the identified genes in gut colonization, we generated isogenic mutants for 9 candidate genes (6 genes required for gut colonization and 3 genes whose mutants were enriched in the gut; Fig. S3 and Fig. 5) using the Lambda Red recombination system (Fig. S7; see also supplemental methods, Text S2, for details at doi.org/10.6084/m9.figshare.7063823) (22). We selected genes located in the same operon (*cyoA/B*, *tamA/B*, *yqjA*/IG_gene_0614), genes with potentially pharmacologically targetable functions (e.g., stress response-related or membrane-associated genes; *gltB*, *ybgF*, *hemN*), and genes that were highly enriched in multiple animals (*fhlA*, gene_2133, gene_2182). For intergenic_gene_3858 (Fig. S6A), we deleted the downstream *ybgF* gene, which encodes a Tol-Pal system protein involved in maintenance of the outer membrane integrity (23). We also generated mutants for *fimD* and *kpjC*, which were previously reported to be involved in gut colonization by uropathogenic E. coli (UPEC) UTI89 or K. pneumoniae LM21 (24, 25) but were not detected in our INSeq screening. Two mutants with a defect in anaerobic *in vitro* growth (gene_0158 [*gpml*] and gene_4942 [*nrdD*]: Fig. S6B) were included as controls. All the mutants, except the two *in vitro* controls, were comparable to the wild type in both aerobic and anaerobic *in vitro* growth, although the Δ*cyoB* and Δ*yqjA* mutants grew slightly slower than the wild type in aerobic culture by 24 h, while Δ*fimD* and Δ*fhlA* mutants grew slightly better than the wild type in anaerobic culture (Fig. S8). To test the ability of the isogenic mutants to densely colonize the intestine, we inoculated antibiotic-treated mice with a 1:1 mixture of the wild type and each mutant strain and tracked their colonization levels for 4 weeks (Fig. 6 and Fig. S9). As predicted, Δ*tamA*, Δ*hemN* Δ*gltB*, Δ*yqjA*, and Δ*ybgF* mutants were defective in gut colonization (Fig. 6A and C). Particularly, Δ*tamA* and Δ*hemN* mutants showed dramatic defects in gut colonization, resulting in 4 to 5 log_10_ loss in 7 days; Δ*gltB* mutant also showed a significant and persistent defect, resulting in 4 log_10_ loss in 28 days (Fig. 6A). Introduction of the complementary plasmids encoding the deleted gene operons (see supplemental methods, Text S2, posted at doi.org/10.6084/m9.figshare.7063823 for details) significantly compensated for the colonization defects observed in Δ*tamA* and Δ*hemN* mutants (Fig. 6B)—these two loci were selected to target, since Δ*tamA* and Δ*hemN* mutants showed the most dramatic defects during early stages of gut colonization, and long-term *in vivo* complementation is challenging in the absence of selective pressure to maintain the complementing plasmid. Δ*fhlA* and Δ*gene_2133* mutants showed enhanced fitness as expected, whereas the Δ*gene_2182* mutant was slightly defective at days 4 and 7 (Fig. 6D). The Δ*cyoB* mutant did not show a fitness change (Fig. 6C). Although the competitive index of Δ*fimD* and Δ*kpjC* mutants trended toward decreased fitness, the decrease did not achieve statistical significance (Fig. S10). Of note, since we only tested a small number of animals, particularly for the Δ*fimD* mutant, statistical significance might be achieved with more animals.

10.1128/mBio.02663-18.7 FIG S7 Colony PCR of isogenic mutants to confirm genotype, purity, and loss of pKD46_*aadA*. (A) Colony PCR of transformants to screen for deletion mutants. Wherever possible, four colonies were picked for each gene and genotyped by PCR using a forward primer that binds upstream of the gene ORF and two reverse primers that bind either to the gene ORF to detect a wild type or to the rifampin cassette to detect a mutant. The reverse primers were designed to have larger PCR products for mutants to sensitively detect wild-type contaminants. Lanes: 1 to 4, distinct colonies; C, water controls. (B) Confirmation of the purity of isolated mutants. Mutants identified in the initial screening in panel A were restreaked on fresh plates to isolate pure mutants, and absence of wild-type contaminants was confirmed by colony PCR. (C) Confirmation of the absence of pKD46_*aadA* in isolated mutants. MH258 carrying pKD46_*aadA* (used for electroporation) was used as a positive control (Con, +). Download FIG S7, PDF file, 1.1 MB. Copyright © 2019 Jung et al. 2019 Jung et al. This content is distributed under the terms of the Creative Commons Attribution 4.0 International license.

10.1128/mBio.02663-18.8 FIG S8 Competitive *in vitro* growth study with isogenic mutants. The same mutants used for the mouse study in Fig. 6 were tested for aerobic and anaerobic *in vitro* growth in a competition with a wild-type strain. Mixtures (1:1 mixtures) of the wild type and each mutant strain (∼5 × 10^2^ CFU of each strain per ml) were inoculated into BHI medium and incubated at 37°C either aerobically or anaerobically. At an exponential (∼6 to 7 h postinoculation) and stationary (24 to 26 h p.i.) growth phases, serial dilutions of the cultures were plated on BHI plates with or without rifampin. Mean competitive index (CI) ± SEM for each mutant is shown in a log scale. *, *P* < 0.05 by one-sample *t* test. Download FIG S8, PDF file, 0.8 MB. Copyright © 2019 Jung et al. 2019 Jung et al. This content is distributed under the terms of the Creative Commons Attribution 4.0 International license.

10.1128/mBio.02663-18.9 FIG S9 Actual CFUs in the competitive colonization study in Fig. 6 and Fig. S10. Median CFU/g feces is shown with a 95% confidence interval. Download FIG S9, PDF file, 0.7 MB. Copyright © 2019 Jung et al. 2019 Jung et al. This content is distributed under the terms of the Creative Commons Attribution 4.0 International license.

10.1128/mBio.02663-18.10 FIG S10 Competitive colonization study with reference and control mutant stains (Δ*fimD* and Δ*kpjC*; Δ*gpml* and Δ *nrdD*). Mean competitive index (CI) ± SEM for each mutant is shown in a log scale. Statistical significance by one-sample test: *, *P* < 0.05; **, *P* < 0.01; ***, *P* < 0.001. Download FIG S10, PDF file, 0.3 MB. Copyright © 2019 Jung et al. 2019 Jung et al. This content is distributed under the terms of the Creative Commons Attribution 4.0 International license.

Conserved genes have a greater potential for therapeutic intervention. Therefore, we compared the genomes of K. pneumoniae MH258 and other K. pneumoniae clinical strains belonging to different MLSTs (multilocus sequencing types) to assess conservation of the colonization factors identified in this study (Table 1; see also Fig. S11 posted at doi.org/10.6084/m9.figshare.7063823). With the exception of two hypothetical proteins (gene_1438 and gene_1858), all genes associated with intestinal colonization were highly conserved in all K. pneumoniae strains.

**TABLE 1:**
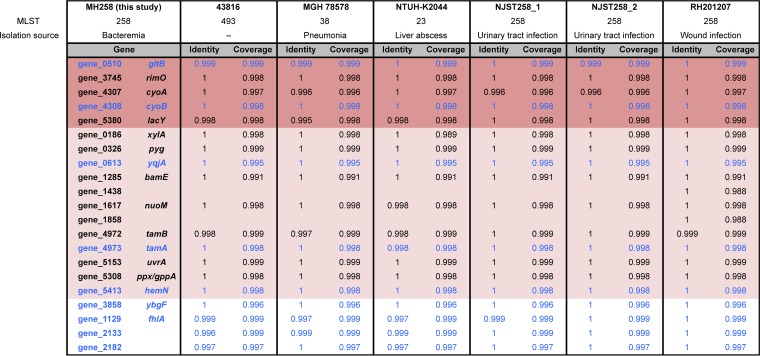
The identified colonization factors are highly conserved among K. pneumoniae clinical strains^*a*^

a To assess conservation of the genetic factors identified in this study, the genomes of K. pneumoniae MH258 (http://www.ebi.ac.uk/ena/data/view/PRJEB31265), 43816 (GenBank accession no. CP009208), MGH78578 (CP000647), NTUH-K2044 (AP006725), NJST258_1 (CP006923.1), NJST258_2 (CP006918.1), and RH201207 (LT216436) were compared using the Proteome Comparison tool of PATRIC 3.5.27 (https://www.patricbrc.org/) (50). Bidirectional protein sequence identity (Identity) and sequence coverage (Coverage) are shown. Genes presented in Fig. 5 are highlighted with the same color scheme as in Fig. 5 (dark pink and light pink background); genes whose isogenic mutants were tested for a competitive colonization study (Fig. 6) are indicated in blue type.

## DISCUSSION

Bacteria belonging to the Enterobacteriaceae family, such as Klebsiella pneumoniae, are often long-term inhabitants of the gastrointestinal tract. Most healthy individuals harbor low densities of *Klebsiella* species in their lower intestinal tract, and the commensal microbiota suppresses their expansion. Depletion of the commensal microbiota can result in dramatic expansion of Klebsiella pneumoniae, as seen in Fig. 1, demonstrating their ability to thrive in and densely colonize the intestinal tract under conducive conditions. Our study demonstrates that a number of different genes that are essential for early and late stages of dense gut colonization encode inner/outer membrane proteins or proteins involved in carbohydrate metabolism. Genes in DNA repair/metabolism, glutamate metabolism, and porphyrin metabolism were also identified as contributing to dense colonization. These implicated genes might facilitate K. pneumoniae survival when resources are limited and under a variety of stresses encountered during persistent colonization of the intestine. Surprisingly, genes directly involved in formation of pili/fimbriae, biofilm, or capsule—all of which are well-known virulence factors (17)—were not detected as essential for dense colonization even while the library included many mutants in those genes. It is possible that the stringent criteria we applied to identify genes with persistent fitness changes, while minimizing false-positive rates, excluded virulence factors that make important but relatively minor contributions to gut colonization by ST258 K. pneumoniae. When either *fimD* or *kpjC* was deleted, impacts of the mutations on gut colonization varied significantly, which did not meet the filtering criteria in our INSeq study. In a library of single-insertion mutants, loss of one adhesin might be compensated for by other adhesins, mitigating the impact of the specific mutation. In contrast, genes encoding β-barrel assembly machinery (BAM) complex and translocation and assembly module (TAM) were identified in this study. Recent studies suggested that those proteins have important roles in the assembly of outer membrane proteins, including various types of fimbriae (26–29). In the absence of those proteins, multiple fimbriae might be affected, resulting in more dramatic and consistent defects in gut colonization, as observed in this study.

The kinetics of mutant loss in the competitive colonization studies differed from those observed during library screening. The difference may result from differences in the gut environments in these two circumstances. Library screening involves complex populations, and the wide range of mutants in the library likely influences the fitness of a given mutant, either by direct interaction or indirect changes of available resources. Despite this inherent limitation of the INSeq study design, we identified novel genetic loci that potentially have significant impact on dense colonization of the intestine by CR-*Kp*, and 7 out of 9 isogenic mutants showed expected fitness changes, either defective or enhanced. The roles of *tamA* and *hemN* were further confirmed by complementation study. Considering the high noise levels inherent to high-throughput *in vivo* studies, our prediction of gene essentiality from library screening illustrates the robustness of this approach.

How the identified genes contribute to gut colonization by ST258 K. pneumoniae will require further study. Persistent, high-density colonization of the intestinal lumen is a complex process involving many microbial and host factors. Colonizing bacteria must utilize available nutrients to persist and proliferate sufficiently to make up for expulsive losses. Colonizing bacteria also must resist antimicrobial molecules released by the host and other cocolonizing competitors. Adding to the complexity, resources and stresses that bacteria encounter likely differ during the early and later stages of dense colonization. Attachment to the host surfaces to resist expulsion by peristaltic waves is another requisite for bacteria to persist in the gut. Thus, at a minimum, genes associated with nutrient uptake, resistance to host defenses, and adhesion are likely to contribute to persistence in the gut. A recent study showed that deletion of *dedA* (a *yqjA* homolog) restored susceptibility of ST258 K. pneumoniae to colistin (30). Other studies suggest roles of *gltB* and *hemN* in stress responses, including settings where resources are limited (31–35). It is also plausible that the identified genes, including the ones encoding hypothetical proteins, have yet-to-be defined roles. With better understanding of the underlying mechanisms, the identified genes might present potential targets to limit dense colonization of the intestine by ST258 K. pneumoniae.

We expected that the mutant population in the library would be quite skewed through potential bottlenecks en route to the colon. To our surprise, most mutants reached the colon and colonized it by day 1; the diversity was preserved, and only a few mutants were significantly depleted. However, by day 4, when the density of the bacteria reached the maximum, the diversity of the mutant population was greatly reduced. We also observed that a small subset of mutants had markedly increased fitness at this point. It is likely that marked expansion of some mutants led other mutants to fall below detection levels but not to their complete loss. This idea is supported by the fact that after day 4, the diversity of mutant populations gradually recovered and stabilized by day 14. While the simulation-based normalization of input data by the ARTIST pipeline corrected potential sampling errors due to low sequencing saturation (20), much deeper sequencing would likely detect low-abundance mutants.

Mutants with enhanced fitness have been previously reported in diverse settings (36–39). For gene_2182, gene_3185, and gene_5027, enrichment of the mutants may result from a gain of function because only single insertion site mutants showed enhanced fitness. If it resulted from a loss of function, mutants at the other sites should have gained a similar fitness enhancement. In contrast, enrichment of multiple mutants in the same gene or genes in the same pathways suggests that the identified genes likely have adverse effects in gut colonization. These can be energy costs for gene expression, while the gene products are dispensable at least for the moment. Or, the genes might encode targets of the host immune system that directly or indirectly limit intestinal colonization by K. pneumoniae. Although those genes seem costly for the early stages of intestinal colonization, they might play essential roles in the later stages or other environments—which can explain why mutants that were strongly enriched at days 4 and 7 were lost at the later stages, allowing other mutants below the detection limit to recover. A previous study with Pseudomonas aeruginosa also showed that mutants lacking type IVa pili had reduced ability to disseminate although they were enriched in the gut (39).

Several user-friendly tools have been developed to facilitate the analysis of millions of sequencing reads generated in transposon insertion sequencing (20, 40). However, quality and characteristics of sequencing reads differ between studies, and a significant amount of sequence analysis still depends on empirically customized codes. In this study, we examined the impact of different filtering conditions and found significant differences in the final outputs—raising concerns about the reliability of any one given approach. Without validating individual calls using deletion mutants, it is hard to know which condition preserves most true-positive results while minimizing false-positive results. It is also questionable whether one approach would work equally well across different experiments. To retrieve significant genes with higher confidence, read-filtering conditions and parameter settings must be chosen cautiously.

In the development of new therapies for bacterial infection, a desirable feature is low selective pressure for resistance (41). One approach to achieve this is to identify targets that do not reduce survival or growth of the pathogen but instead reduce its ability to persist in the host. Herein we identified genetic factors that are associated with gut colonization but not with growth. Future studies will determine whether the expression of these genes or their products can be blocked in order to reduce the density of intestinal colonization.

## MATERIALS AND METHODS

### Sample collection and 16S rRNA sequencing.

Fecal samples from a patient undergoing allo-HCT at Memorial Sloan Kettering Cancer Center (MSKCC) were collected following an institutional fecal biospecimen collection protocol (12). DNA extraction and 16S rRNA sequencing of fecal samples were performed as described previously (11, 42). Briefly, genomic DNAs were extracted using phenol-chloroform/isoamyl alcohol and 0.1-mm zirconium beads and further purified using QIAamp Mini Spin Columns (Qiagen). The V4-V5 region of the 16S rRNA gene was PCR amplified and sequenced on the Illumina Miseq platform (2x250). The paired-end reads were analyzed using the UPARSE and MOTHUR pipelines (43, 44). Total bacterial loads in each sample were estimated by 16S qPCR (please see supplemental methods, Text S2, posted at doi.org/10.6084/m9.figshare.7063823 for details).

### Bacterial strains and growth conditions.

MH258 is a clinical isolate from MSKCC (18). PIR1 competent Escherichia coli was purchased from Thermo Fisher Scientific. Unless otherwise stated, all the bacteria were grown in Luria-Bertani (LB) broth or on LB agar at 37°C. As appropriate, the following antibiotics were added to the media: ampicillin (100 μg/ml), streptomycin (50 μg/ml), kanamycin (50 μg/ml), carbenicillin (100 μg/ml), neomycin (50 μg/ml), and rifampin (25 μg/ml).

### Construction of a transposon mutant library.

The transposon vector used in this study (pSAM_Kp2.1) was generated by four modifications of pSAM_Bt (16) as follows. The selection markers in plasmid pSAM_Bt (*ermG* for erythromycin selection and *bla* for ampicillin selection) were replaced with *aadA* from pDB60 (conferring streptomycin resistance) (45) and the kanamycin selection marker from pET-27b(+), respectively. The promoters driving the mariner transposase gene and *aadA* were replaced with the *rpoD* promoter region of strain MH258. All the intermediate plasmids were cloned using In-Fusion Cloning Kits (Clontech) and propagated in One Shot PIR1 chemically competent E. coli (Thermo Fisher Scientific). The final plasmid was fully sequenced and electroporated into E. coli S17 λpir to generate a donor strain. Sequences of all the primers used in this study are listed in Table S4 posted at doi.org/10.6084/m9.figshare.7063823.

For conjugation of the donor strain (E. coli S17 λpir carrying pSAM_Kp2.1) and strain MH258, both strains were grown to an OD_600_ of 0.6 to 0.8, washed twice with PBS, mixed in a 2:1 ratio (donor/recipient), and spotted on 0.45-μm membrane filters (Millipore) on LB plates. After 2 h of incubation at 37°C, the conjugates were suspended in PBS, washed once with PBS, and spread on M9 plates with 50 μg/ml streptomycin. The plates were incubated at 37°C for 14 to 15 h and then flooded with M9 minimal medium containing 20% glycerol to pool colonies. The constructed library was stored in aliquots at −80°C until use.

### Mouse experiments.

Mice were treated with vancomycin (1 g/liter; Novaplus) and metronidazole (1 g/liter; Sigma) in drinking water for 3 days and inoculated with ∼10^8^ CFU of the mutant library in 200 μl PBS by oral gavage. To prepare the inoculum, a frozen aliquot of the mutant library was revived in M9 medium with 50 μg/ml streptomycin for 10 min at 37°C, washed twice with PBS, and diluted in PBS to 5 × 10^8^ CFU/ml. At the time of inoculation, mice were housed singly. The mice were kept on antibiotics throughout the study. Fecal pellets were collected over the next 4 weeks, and half of each pellet was immediately frozen on dry ice and stored at −80°C until used for DNA extraction. To evaluate the colonization levels of the inoculated mutant library, the unfrozen halves of collected fecal samples were suspended in PBS, and 10-fold serial dilutions were plated on M9 agar plates with streptomycin.

For a competitive colonization study, mice were treated with antibiotics as described above and inoculated with 1:1 mixture of the wild type and each mutant strain (∼5 × 10^4^ CFU of each strain) by oral gavage. To determine the density of each strain in feces, serial dilutions were plated on LB plates with or without rifampin (in addition to carbenicillin and neomycin). The wild-type level was deduced from differences in CFU on those plates; the mutant level was determined from CFU on the plates containing rifampin. The competitive index (CI) was calculated as a ratio of mutant CFU to wild-type CFU normalized to the input ratio.

Wild-type C57BL/6 mice were purchased from the Jackson Laboratory, and 6- to 8-week-old female mice were used for the study. All mice were maintained under specific-pathogen-free conditions at the Memorial Sloan Kettering Research Animal Resource Center. All animal protocols were approved by the Institutional Animal Care and Use Committee of MSKCC.

### *In vitro* screening.

Approximately 10^8^ CFU of the mutant library, prepared as described above, was inoculated into 1 liter of brain heart infusion (BHI) medium and incubated at 37°C either aerobically or anaerobically. The anaerobic condition in a chamber (Coy Laboratory Products) with 2.8 to 4.0% hydrogen was maintained using 7.5% hydrogen/5.0% CO_2_/88.5% nitrogen gas mixture. As the cultures reached early stationary phase (OD_600_ of 0.8 to 1.0), they were sampled for DNA extraction and passaged to fresh BHI medium at 10^8^ CFU/liter.

### Preparation of DNA library and sequencing.

Genomic DNAs were extracted as stated above, and DNA libraries were prepared as described previously (46). Briefly, transposon insertion sites were amplified using 0.5 μg of RNase-treated genomic DNA and a biotinylated primer that binds within the transposon (see Table S4 posted at doi.org/10.6084/m9.figshare.7063823). The PCR products were purified using Dynabeads M-280 streptavidin (Thermo Fisher Scientific), digested with MmeI (New England Biolabs), and ligated to sequencing adaptors with distinct barcodes. After a final PCR amplification, the DNA libraries were purified using QIAquick gel extraction kit (Qiagen) and sequenced on Illumina Hiseq 2500 (1x50, Rapid Run).

### Sequencing data analysis.

Using Cutadapt (47), low-quality bases were removed, and transposon sequences were trimmed off. After filtering out reads shorter than 30 bp, the retrieved reads were binned by sample-specific barcodes and aligned to the MH258 genome with Bowtie2 (48). Read counts for each insertion site were tallied and analyzed using the ARTIST pipeline (20). For the MWU analysis, a *P* value cutoff of 0.001 and reproducibility threshold of 0.9 were used; the HMM refinement was skipped. The final gene essentiality assignments for each sample were further analyzed using custom R scripts. Please also see supplemental methods, Text S2, posted at doi.org/10.6084/m9.figshare.7063823 for details of the read filterings prior to the ARTIST MWU analysis.
