# Supplementary figures and images for: Genome-Wide Screening for Enteric Colonization Factors in Carbapenem-Resistant ST258 Klebsiella pneumoniae
Source: mBio. 2019 Mar 12;10(2):e02663-18. doi: 10.1128/mBio.02663-18 (PMC6414703; doi:10.1128/mBio.02663-18)

Figure S1

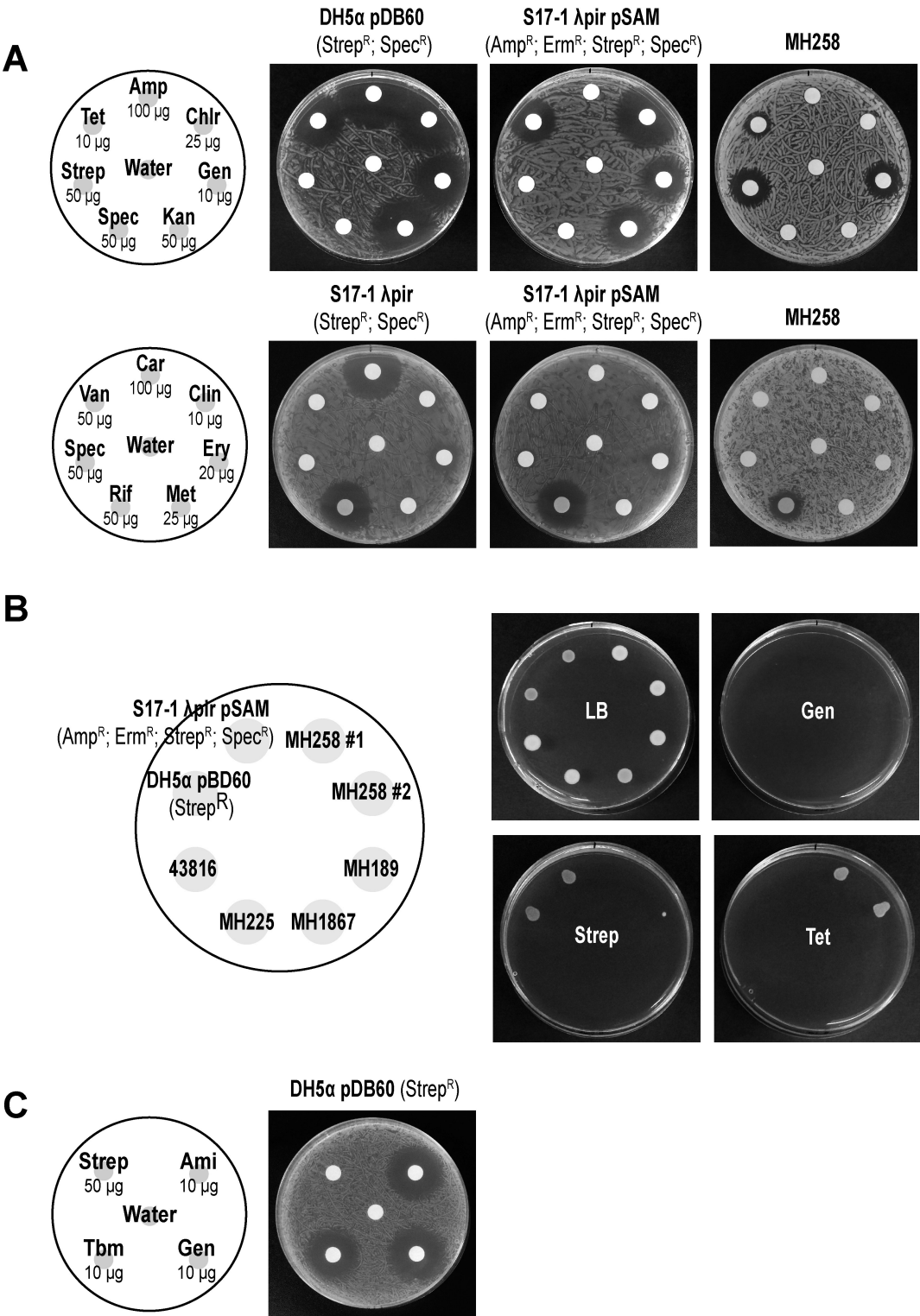

Supplement: FIG S1 [file mBio.02663-18-sf001.pdf]

Figure S2

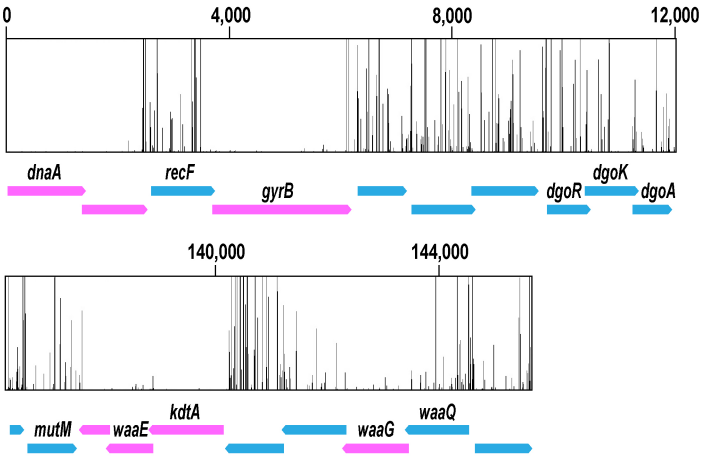

Supplement: FIG S2 [file mBio.02663-18-sf002.pdf]

Figure S3

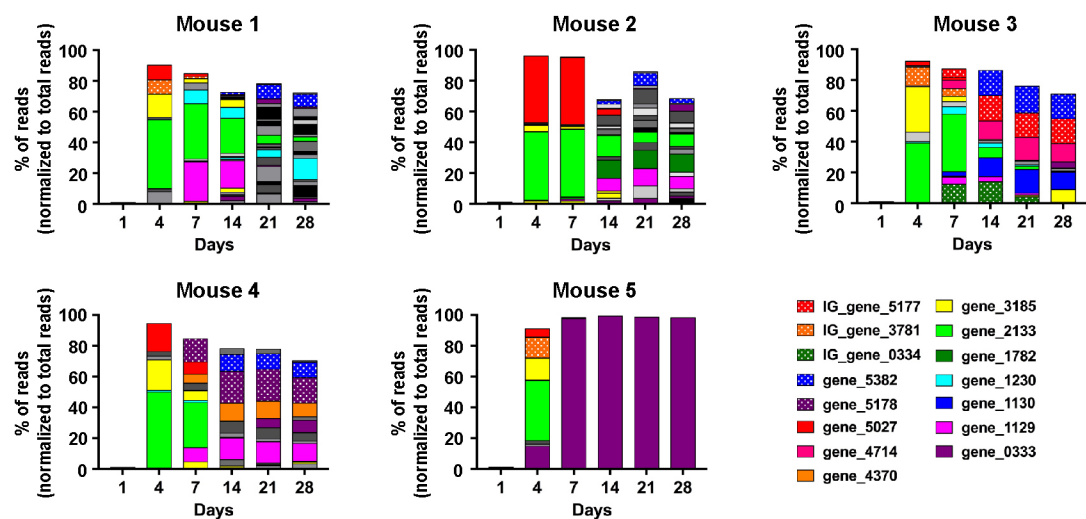

Supplement: FIG S3 [file mBio.02663-18-sf003.pdf]

# gene\_0333

Figure S4(A)

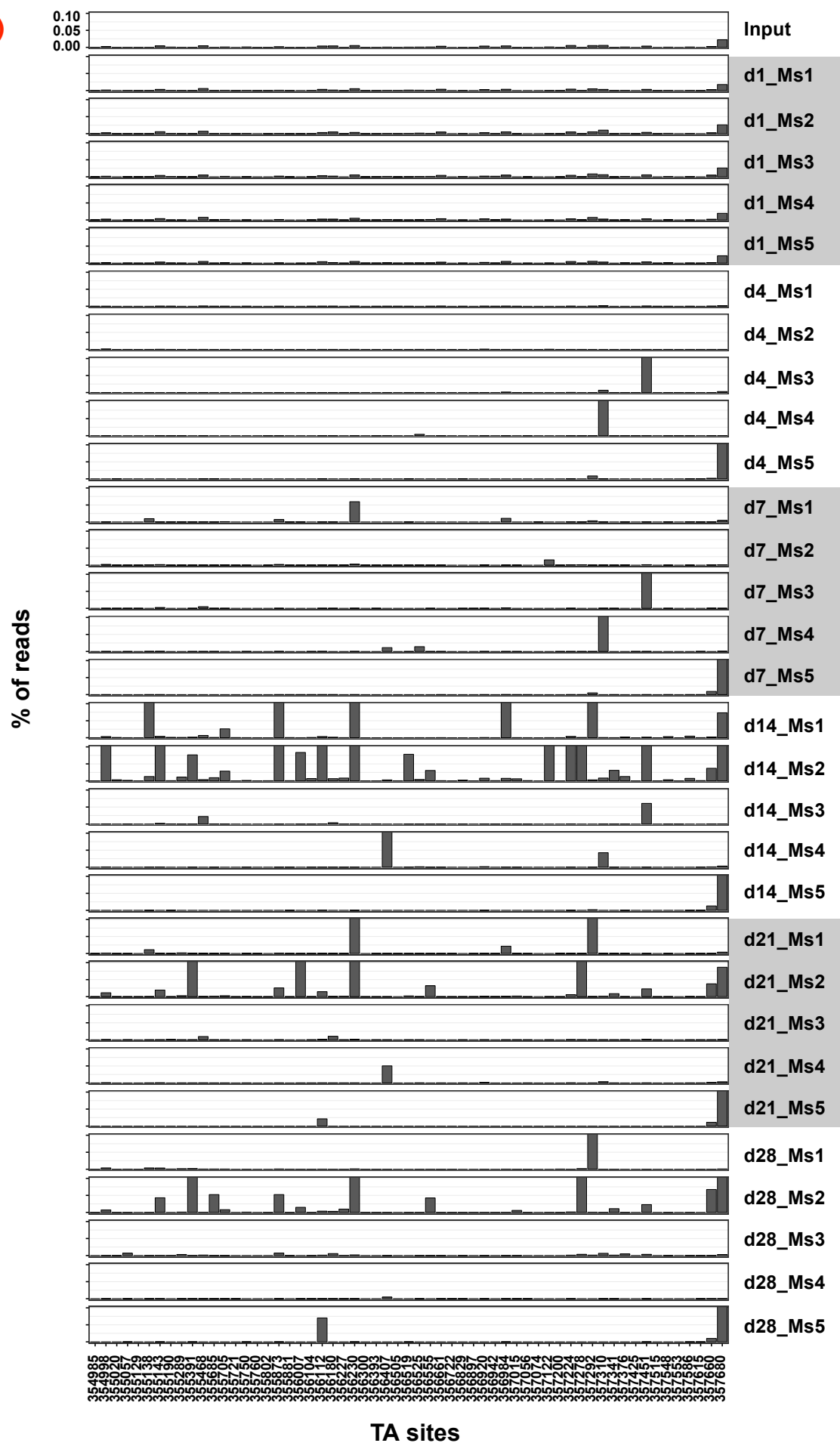

# gene\_1129

Figure S4(B)

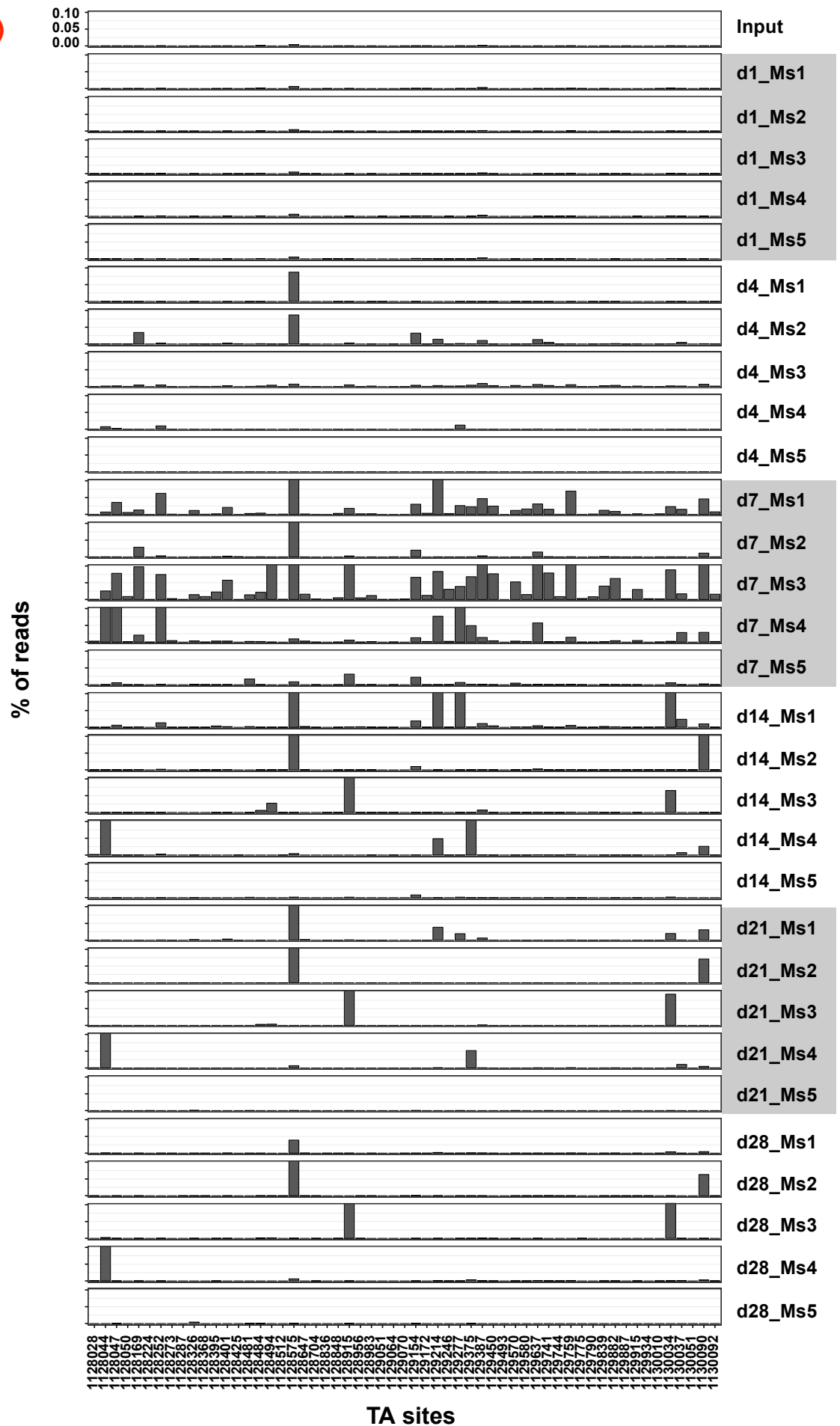

# gene\_2133

Figure S4(C)

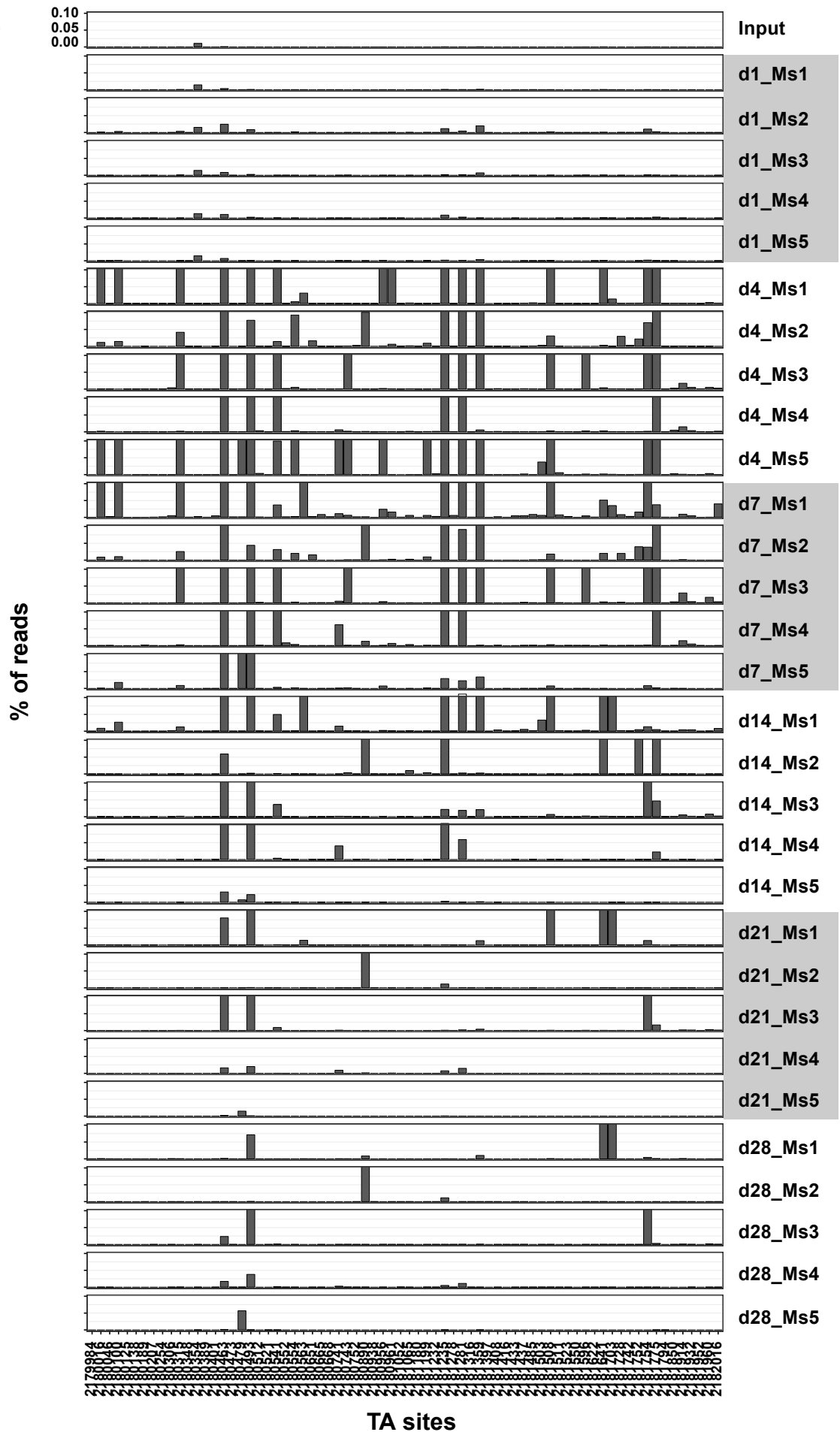

# gene\_5382

Figure S4(D)

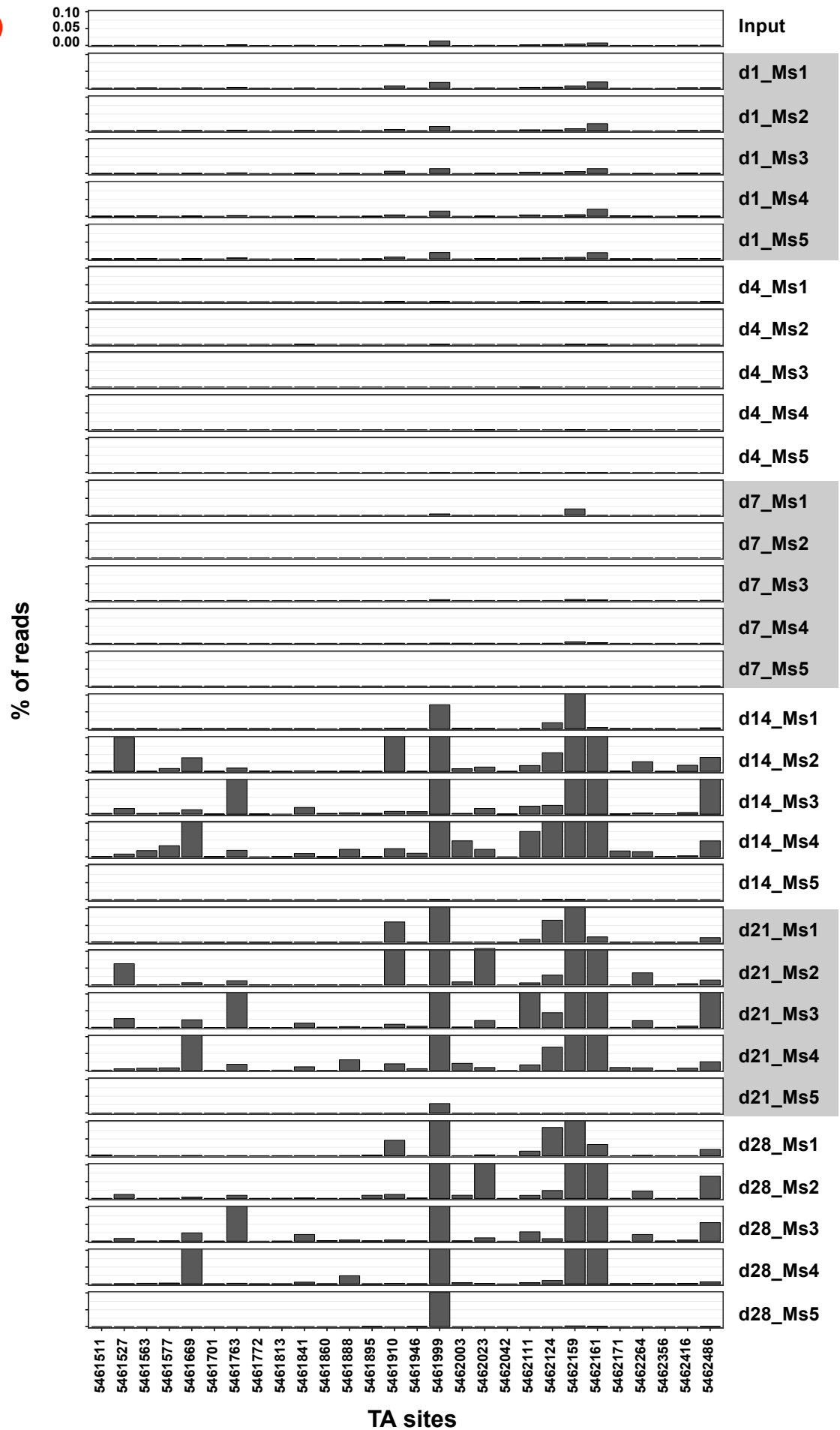

Figure S4(E)

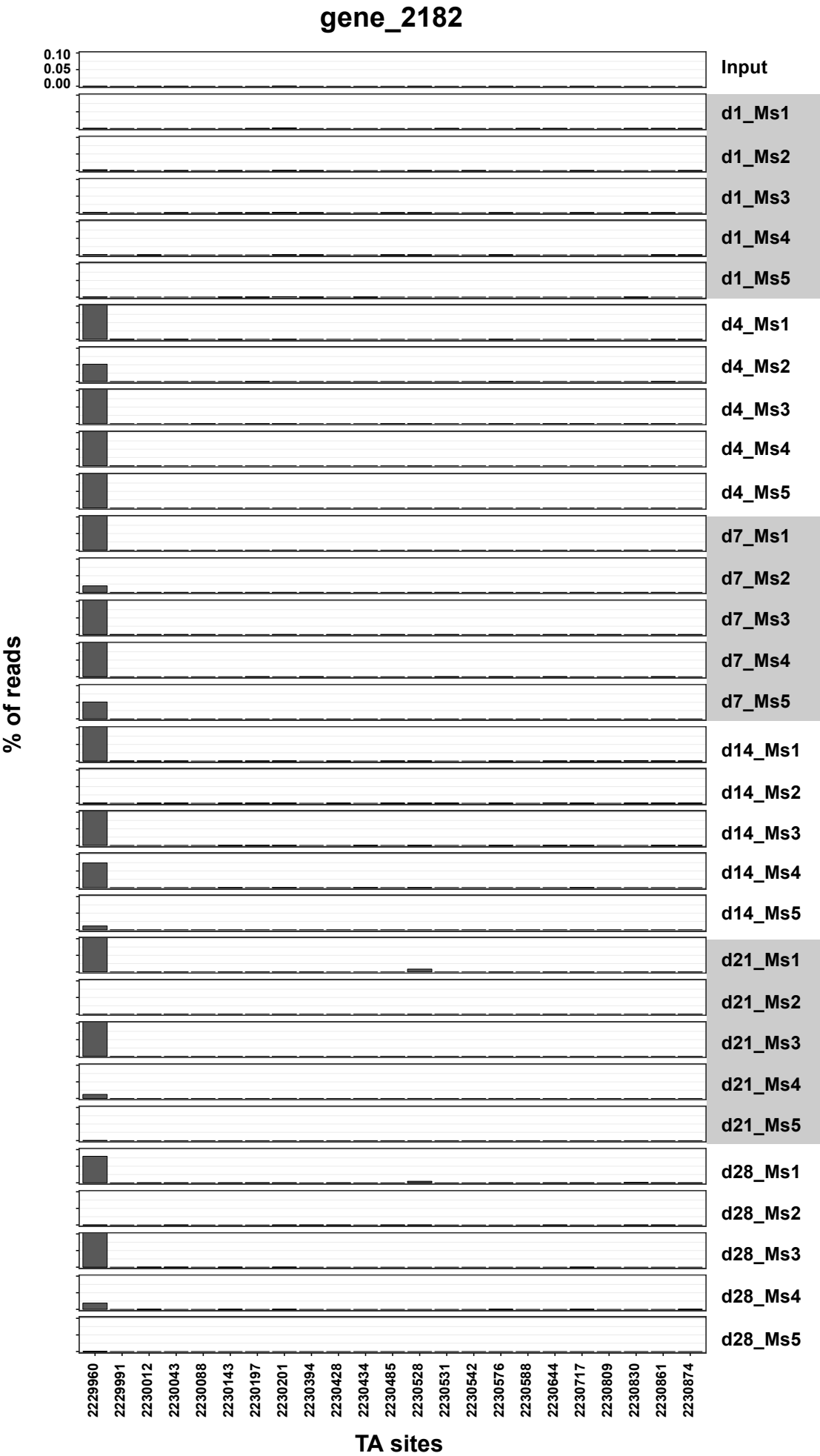

### Figure S4(F)

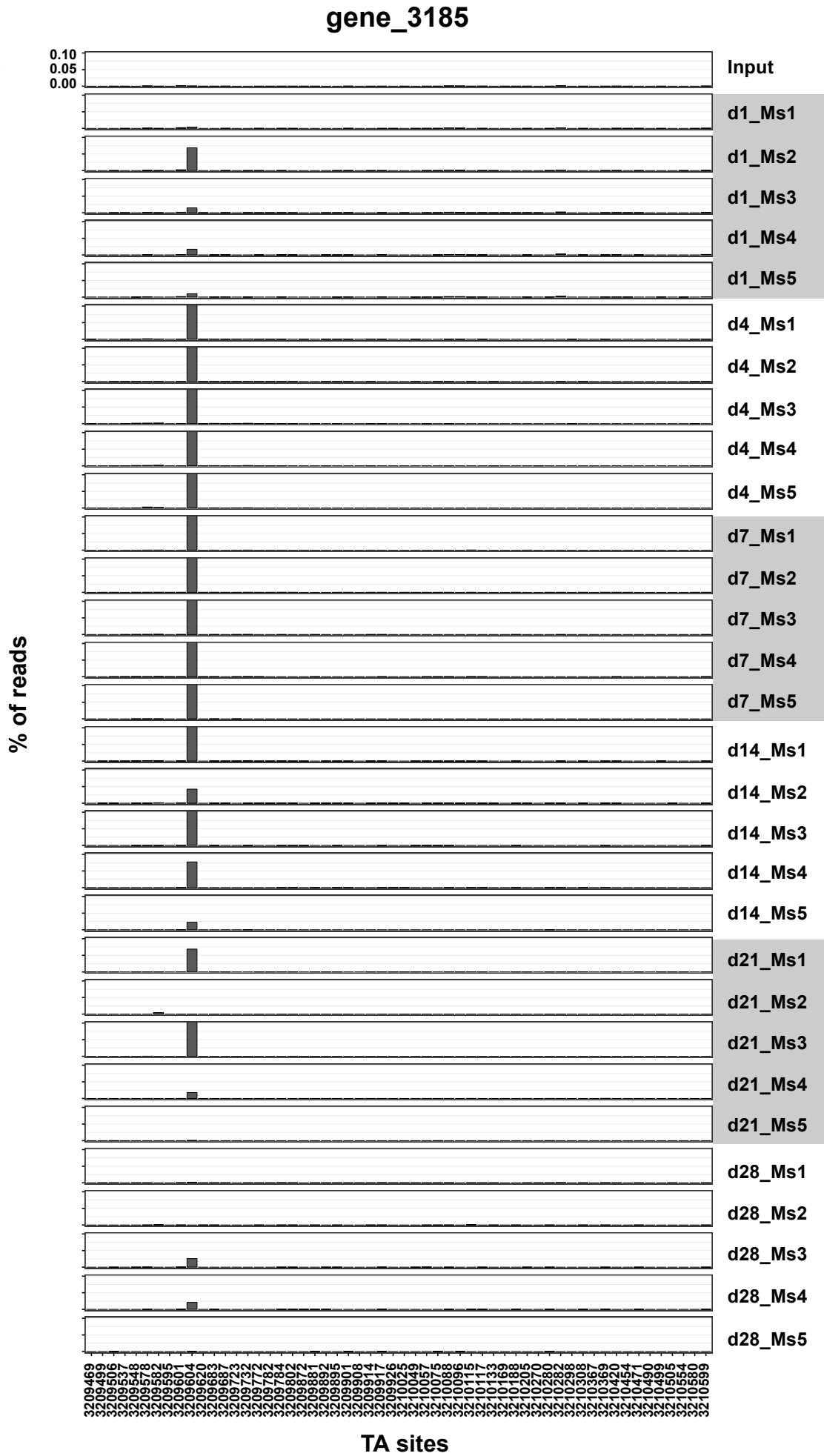

Figure S4(G)

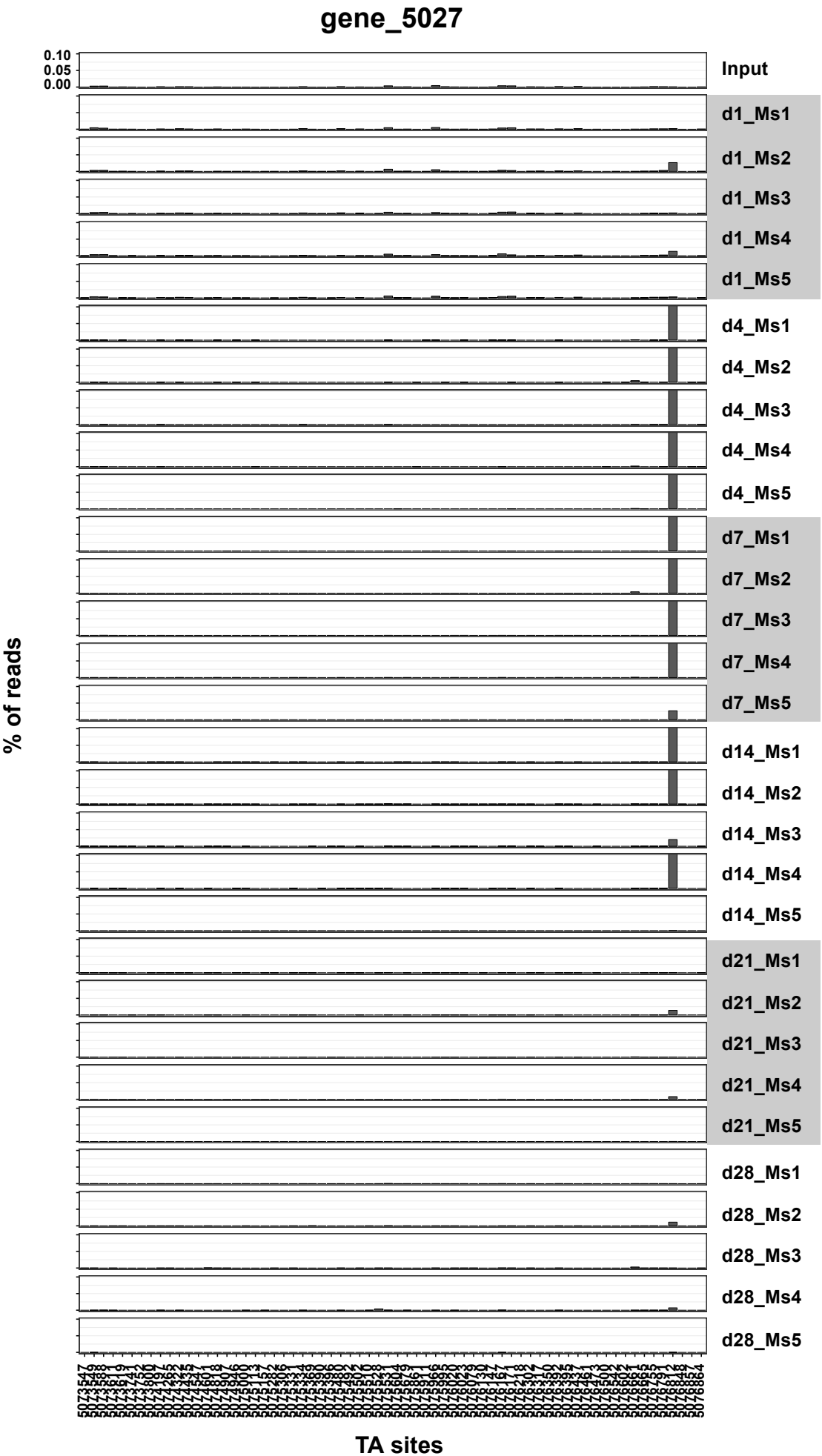

Supplement: FIG S4 [file mBio.02663-18-sf004.pdf]

Figure S5(A)

A

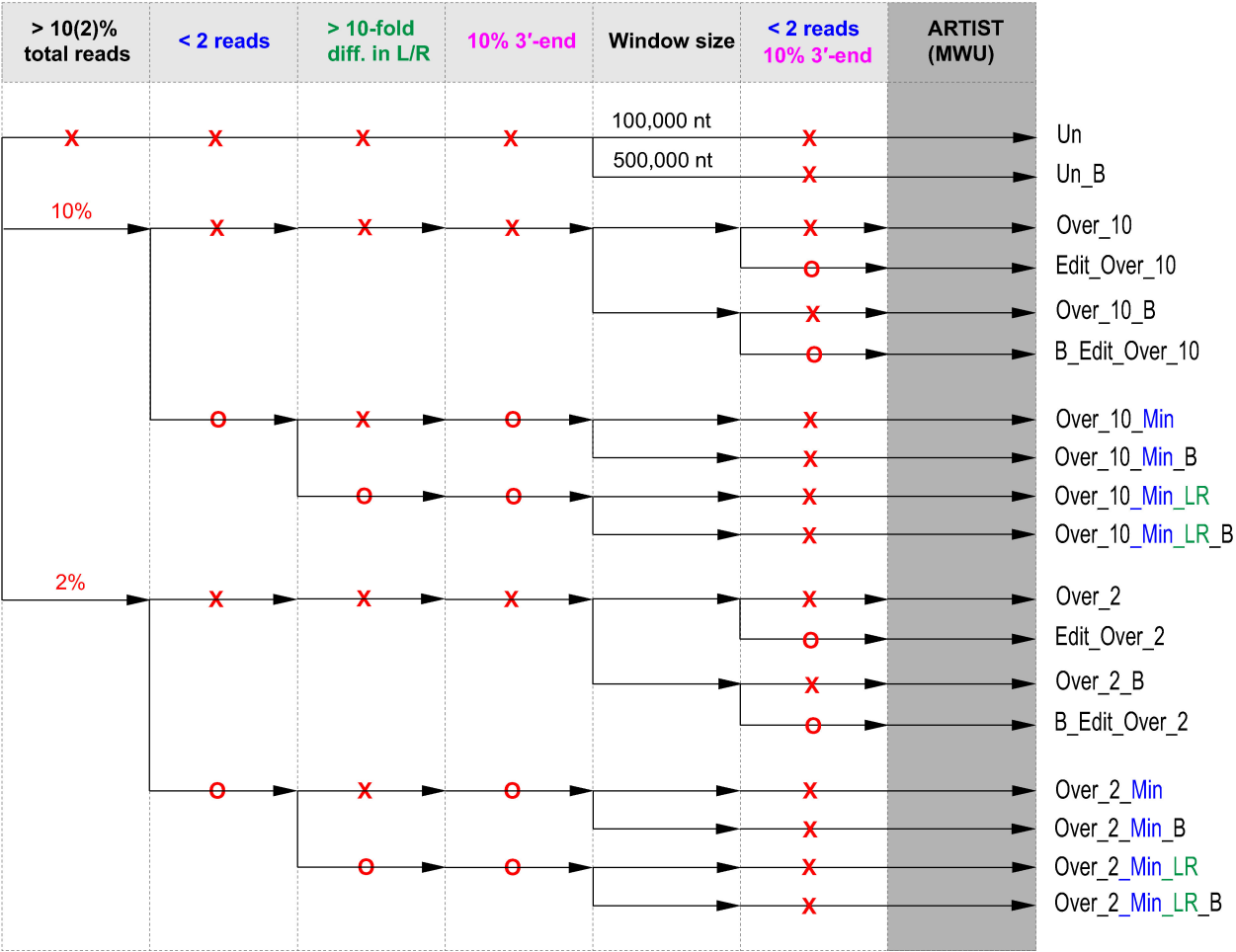

Figure S5(B)

B

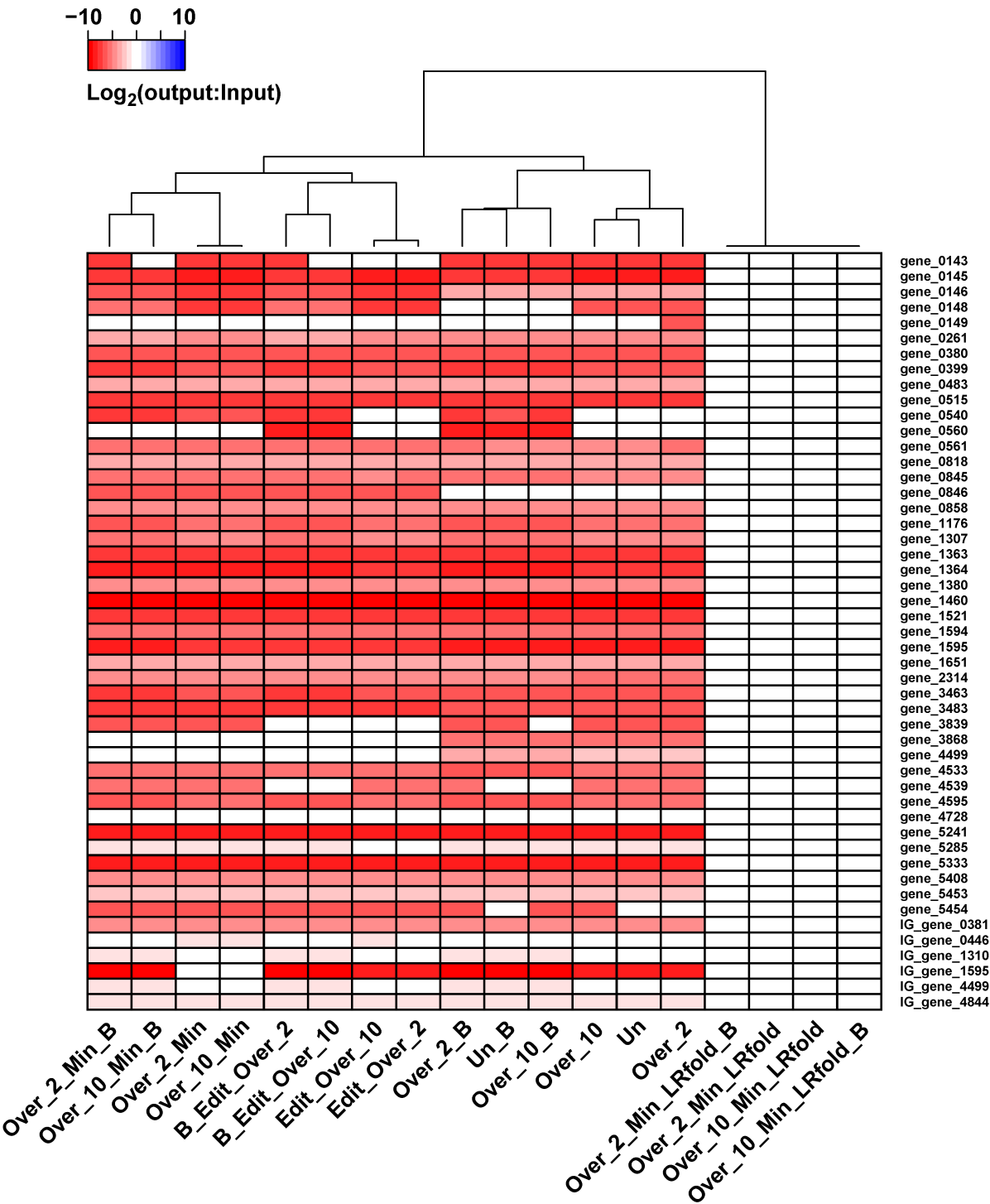

Figure S5(C)

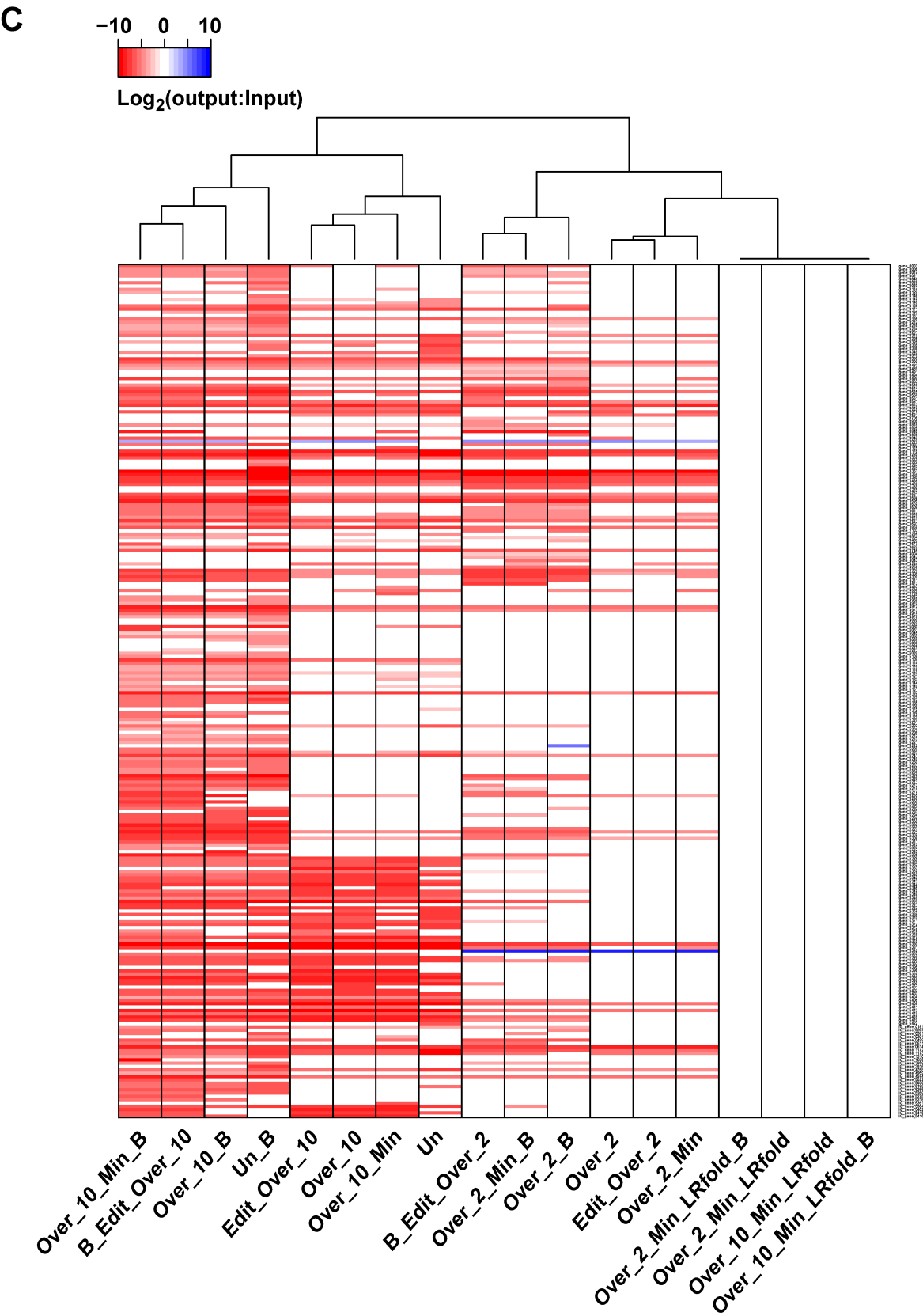

Supplement: FIG S5 [file mBio.02663-18-sf005.pdf]

Figure S6(A)

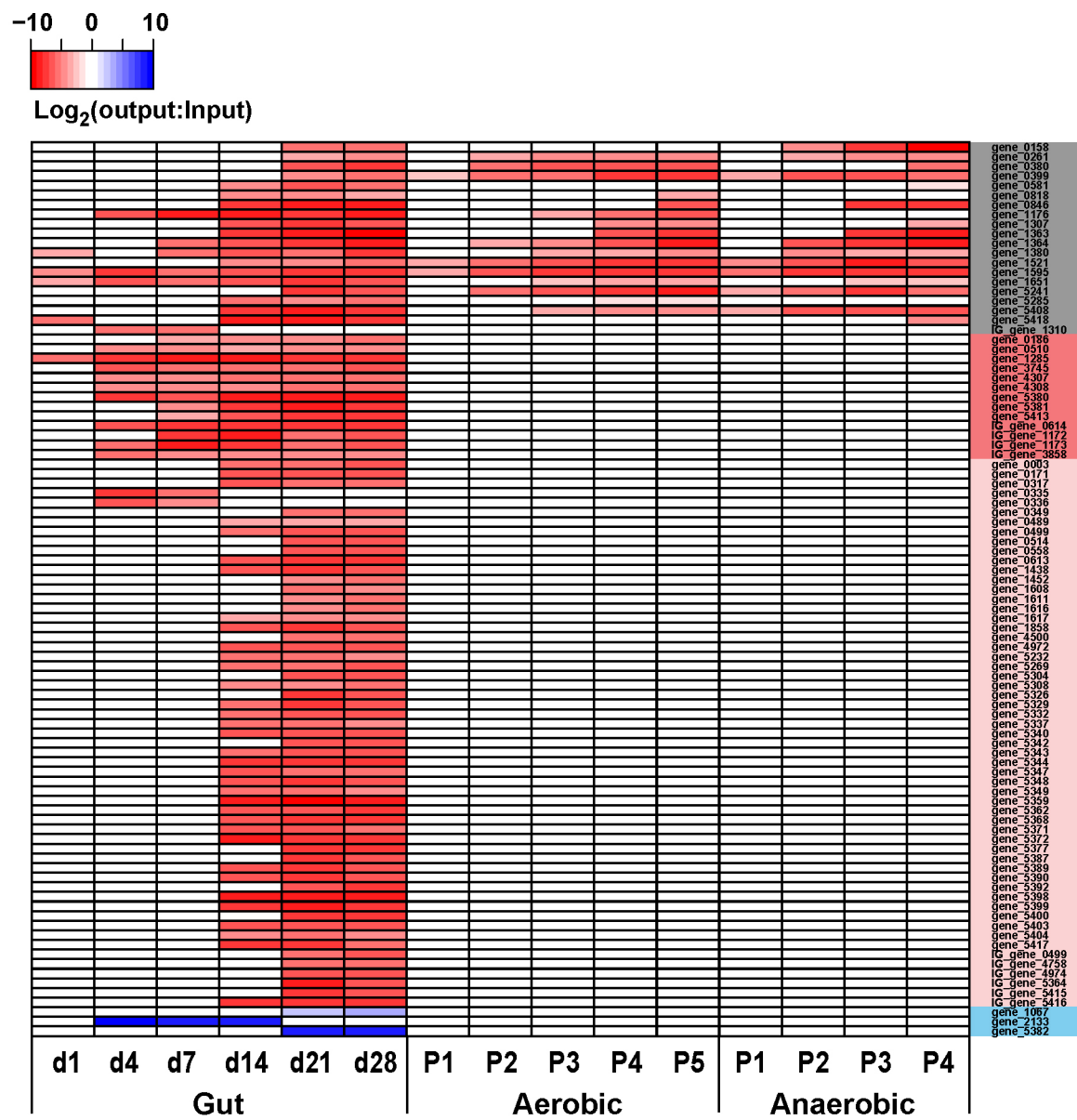

Figure S6(B)

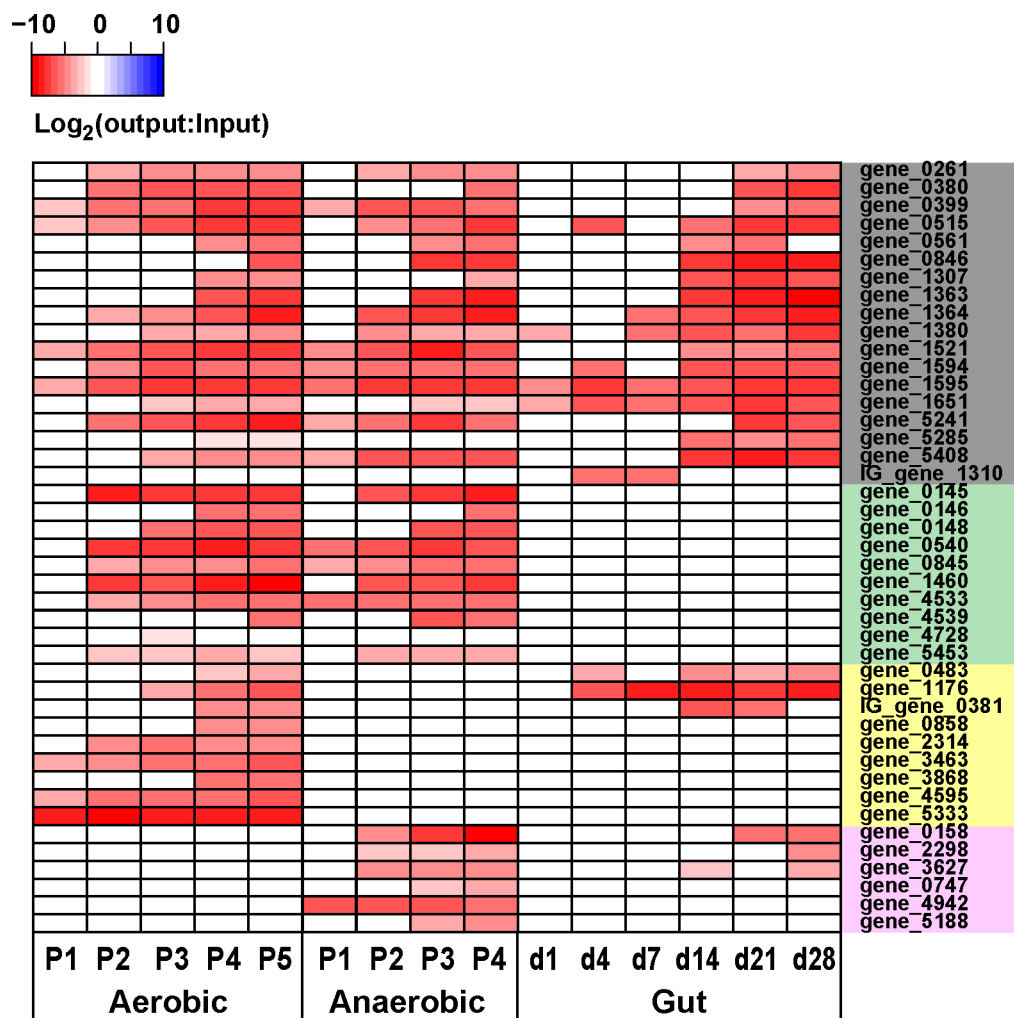

Supplement: FIG S6 [file mBio.02663-18-sf006.pdf]

Figure S7

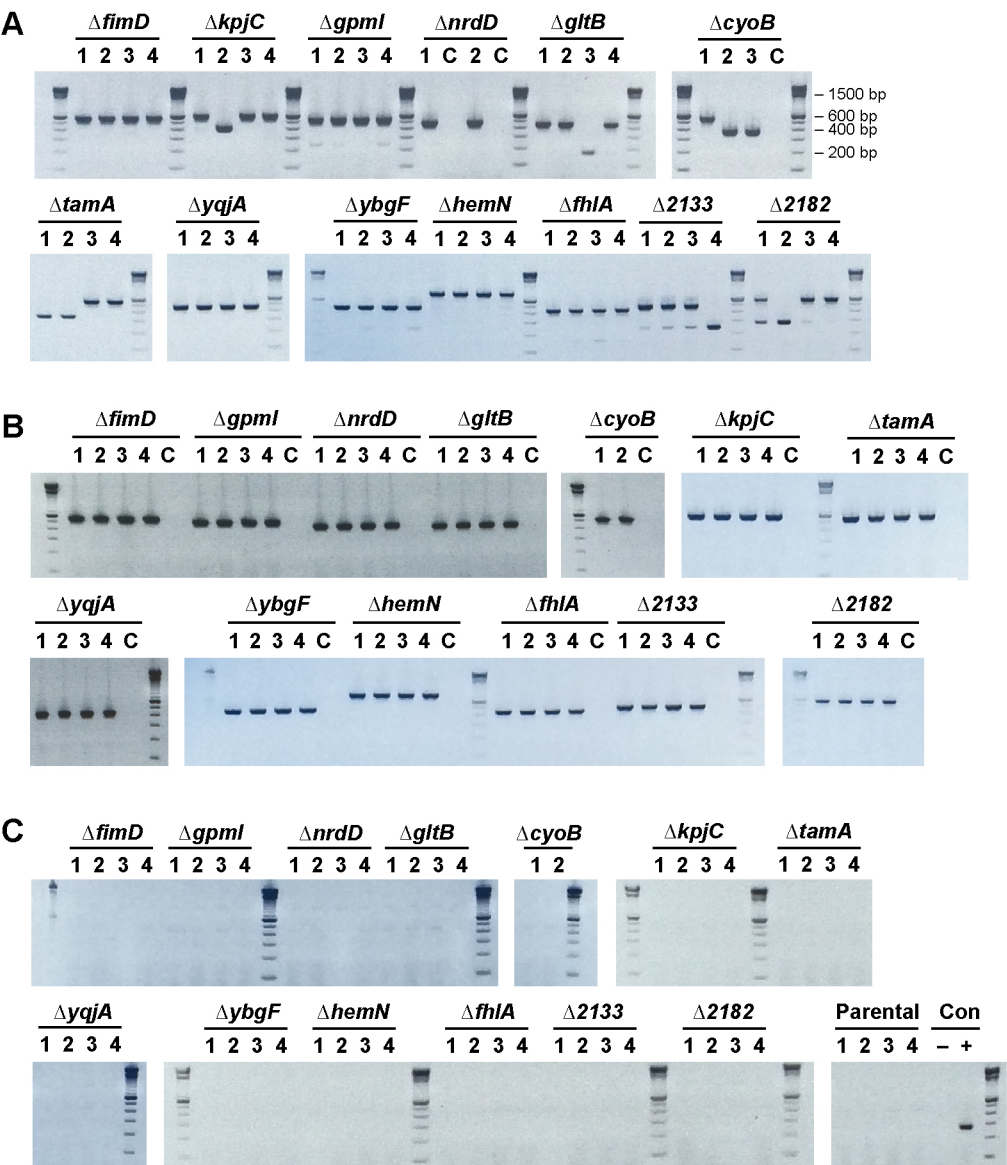

Supplement: FIG S7 [file mBio.02663-18-sf007.pdf]

Figure S8

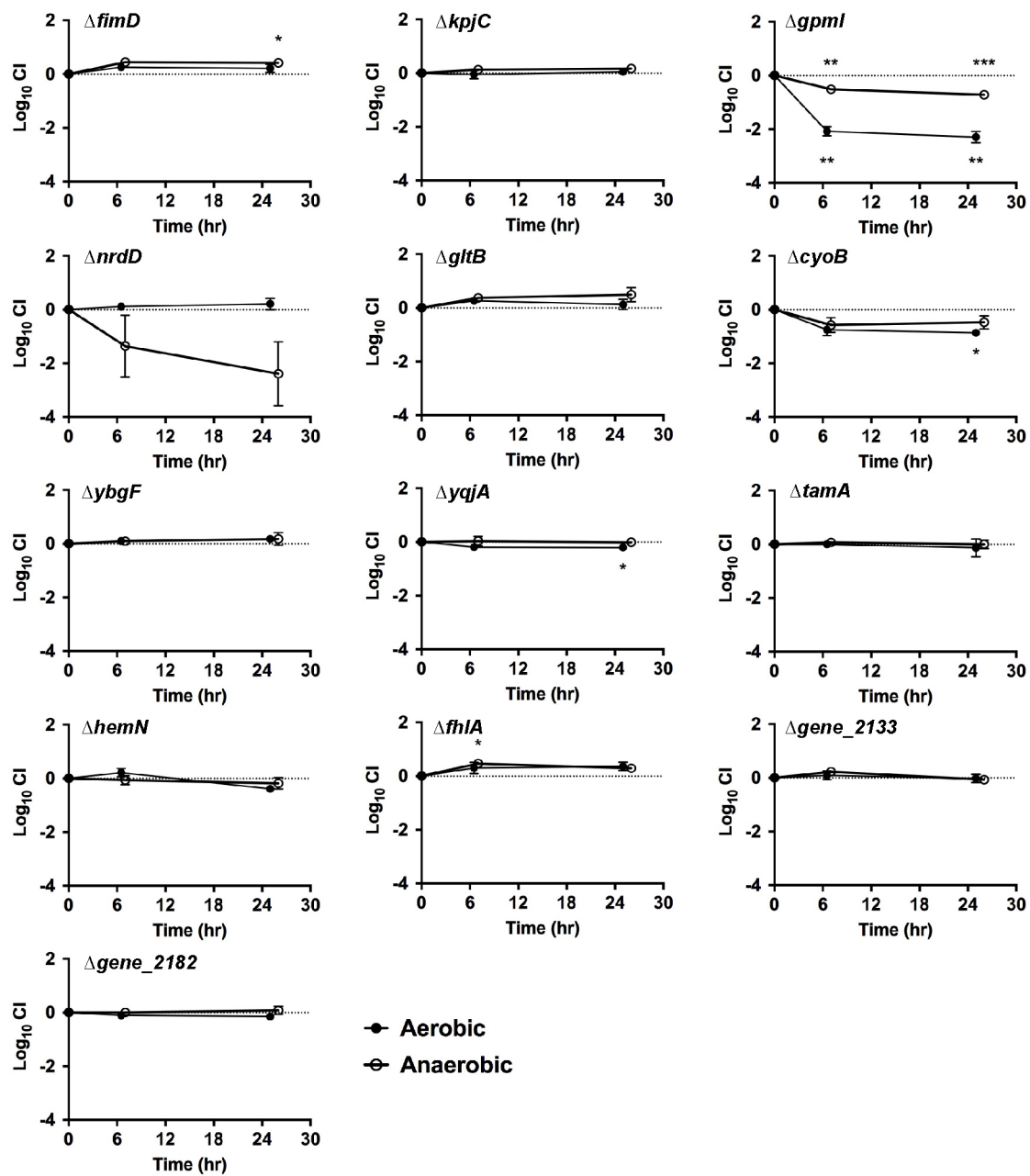

Supplement: FIG S8 [file mBio.02663-18-sf008.pdf]

Figure S9

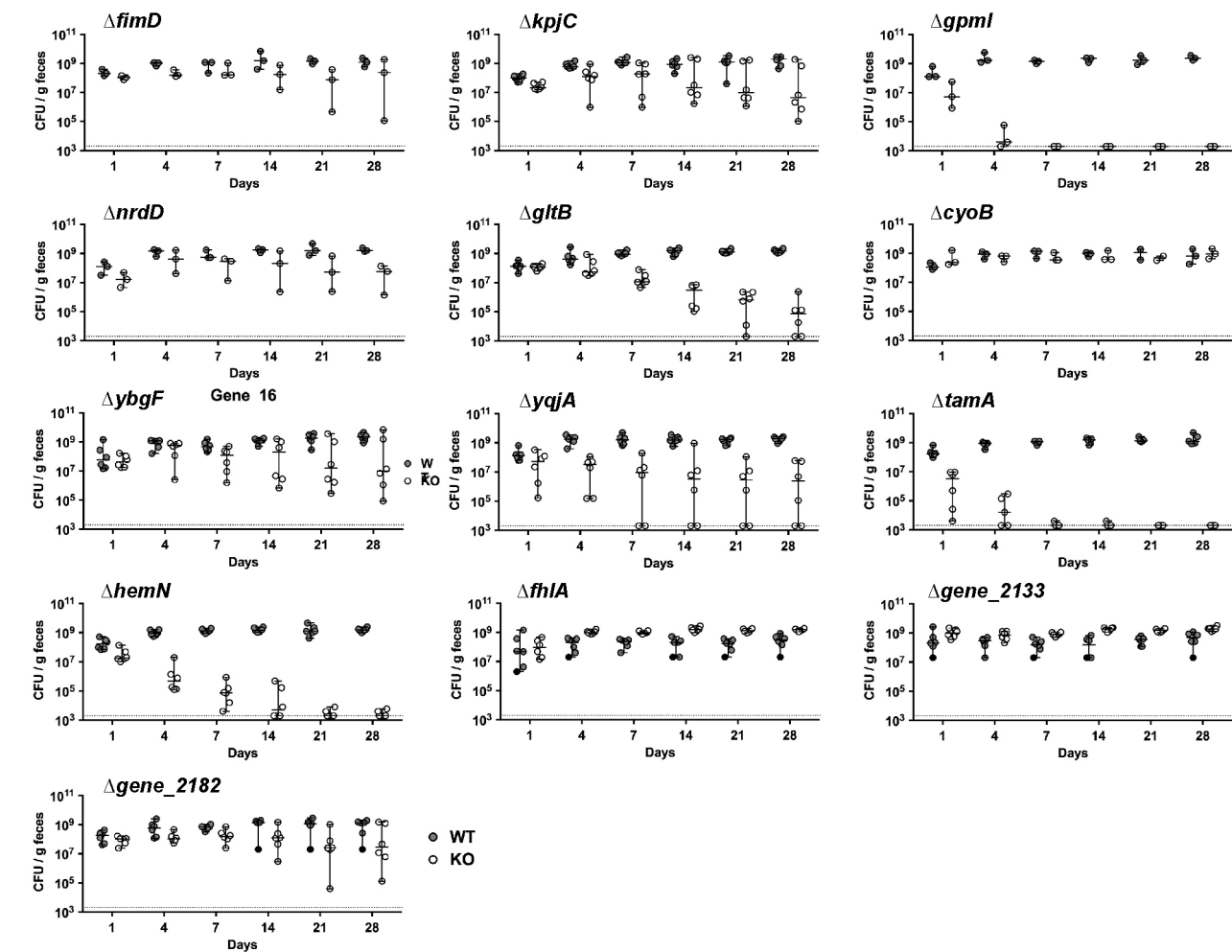

Supplement: FIG S9 [file mBio.02663-18-sf009.pdf]

Figure S10

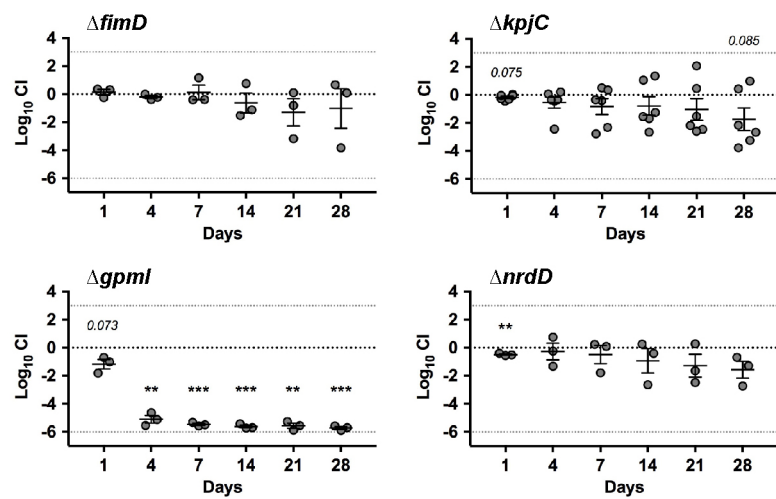

Supplement: FIG S10 [file mBio.02663-18-sf010.pdf]
